# Bifurcation analysis of hepatitis B virus with non-cytolytic cure process on infected liver and blood cells

**DOI:** 10.1038/s41598-023-27468-9

**Published:** 2023-04-29

**Authors:** Tesfaye Tefera Mamo

**Affiliations:** Departement of Mathematics, College of Natural and Computational Science, Debre Berhan University, Post Code 445, Debre Berhan, Ethiopia

**Keywords:** Diseases, Infectious diseases, Hepatitis B, Applied mathematics

## Abstract

The phenomenon of bifurcation in disease transmission models has been observed in a number of epidemiological models. The consequence of bifurcation is that the classical requirement of the reproduction number being less than unity becomes only a necessary, but not sufficient, for disease elimination. This paper addresses the problem of finding the causes of bifurcation in standard deterministic models for the spread of HBV diseases with non-Cytolytic cure processes on infected liver and blood cells. The model contains logistic growth of healthy liver and blood cells and non -Cytolytic cure processes of infected cells. I have got that the model exhibits back ward and forward bifurcations with some conditions. The existence of a backward bifurcation is an interesting artifact since this means that the disease cannot be eradicated by simply reducing the value of the basic reproduction number ${R}_{0}$ below 1.This can have important implications on drug therapy protocols, since it sheds light on possible control mechanisms for disease eradication.

## Introduction

Epidemic models have considerably aided in enhancing awareness into infectious disease transmission patterns in host populations, as well as how an infectious disease might be controlled, reduced, and possibly eradicated^1–4^. The functional form of the force of infection, namely the function characterizing the mechanism of disease transmission; and the description of the intervention policy to counter the disease spread are two of the most important components of modeling an infectious disease^5^.

Mathematical modeling of phenomena in applied disciplines results in equations based on one or more factors that can change over a set of values (the parameter space or set).These parameters describe how the environment influences a system, and thus, a well-designed system should be robust. This means that small fluctuations in the parameters do not change its qualitative behavior. However, if the fluctuations become larger, the behavior of a system might change in the sense that the number or stability property of particular solution varies^6^.

Bifurcation occurs if a topologically non-equivalent phase portrait appears when parameters are changed, and the parameter values are called bifurcation values. If one phase portrait is a distorted version of the other, they are topologically equivalent. Bifurcation theory is the mathematical study of changes in the qualitative or topological structure of a given family, such as the integral curves of a family of vector fields, and the solutions of a family of differential equations^7–9^. The biological factors for a specific model are described by system parameters. These parameters determine the eigenvalues that govern the stability of an equilibrium point. As the parameters change to reflect changes in biological conditions, the eigenvalues change along with them. Some of eigenvalues may move from the left to right half of the complex plane. The point at which at least one eigenvalue has zero real-part is called the bifurcation point^10^.

One of the most important aspects of epidemiological modeling is describing how changes in biological processes affect the parameters of infection dynamics at the population level. The "${R}_{0}$ dogma" is an important early outcome of this work that was obtained from simple models^1^. The spectral radius of the next generation matrix is ${R}_{0}$, the fundamental reproductive ratio. If ${R}_{0}>1$, the disease is likely to spread and persist in the host population. Small initial introductions are not sufficiently transmissible to start an epidemic if ${R}_{0}<1$, therefore an endemic illness will eventually go away. Thus, many control policies like vaccination have focused on reaching coverage levels sufficient to reduce ${R}_{0}$ below 1. However, one of the key problems with epidemiological modeling has been figuring out why and when the ${R}_{0}$ rules can breakdown. In particular, some epidemic models can be bi-stable (stable endemic equilibrium point co-exists with a stable disease free equilibrium point for R_0_ < 1). ${R}_{0}<1$ is a sufficient condition for avoiding an epidemic caused by the introduction of a small number of initial cases, but ${R}_{0}<1$ is not a sufficient condition for eradication of the disease once it is endemic. This phenomenon is known as a ‘backward’ bifurcation.

The bifurcation curve, which is the graph of the force of infection in a population as a function of the basic reproductive number ${R}_{0}$, can graphically depict the behavior at a bifurcation. It has been noted^11^ that in epidemic models with multiple groups, it is possible to have a very different bifurcation behavior at ${R}_{0}=1$. There may be multiple positive endemic equilibria for values of ${R}_{0}<1$, and a backward bifurcation at ${R}_{0}$=1. The qualitative behavior of a system with a backward bifurcation differs from that of a system with a forward bifurcation and the nature of these changes has been described in^7^. Since these behavior differences are important in planning how to control a disease, it is important to determine whether a system can have a backward bifurcation.

Simulations of systems of nonlinear differential equations can be performed with numerical methods. When the numerical results are shown, several options are available: time waveforms, phase diagrams or bifurcation diagrams^12^.

As one varies a parameter, fixed points can coalesce with other fixed points, appear, disappear and change their stability. Values of $\left(x; \mu \right);x\in {\mathbb{R}}^{n} \mathrm{and} \mu \mathrm{is set of parametres}$; at which such things occur are called bifurcation points. There are of course many ways which these things can happen and they all depend on the definition of $f\left(x; \mu \right);$ but there are three generic types of bifurcations: Saddle node, Trans critical and Pitchfork. Each of these types of bifurcations has a generic system which exemplifies their salient features.

In this study, healthy liver and blood cells, infected liver and blood cells and free hepatitis B virus are considered. Using non-cytolytic cure processes of infected liver and blood cells, even by transplantation of hyper toxic infected liver of a patient, sometime reinfection is occurring. The insight of this work is why this reinfection is occurring. For this, I considered bifurcation analysis of hepatitis B virus with non-cytolytic cure process on infected liver and blood cells.

## Materials and methods

### Basic concepts of bifurcation analysis

A bifurcation in dynamical systems happens when a minor, gradual change in a system's parameter values results in an unexpected "qualitative" or topological change in the behavior of the system. It has two types:

A. *Local bifurcations*—which can be analyzed entirely through changes in the local stability properties of equilibria, periodic orbits or other invariant sets as parameters cross through critical thresholds.
B. *Global bifurcation*—which often occur when larger invariant sets of the system ”collide” with each other, or with equilibria of the system. They cannot be detected purely by a stability analysis of the equilibria (fixed or equilibrium points).

### Saddle node bifurcation

**Figure 1:**
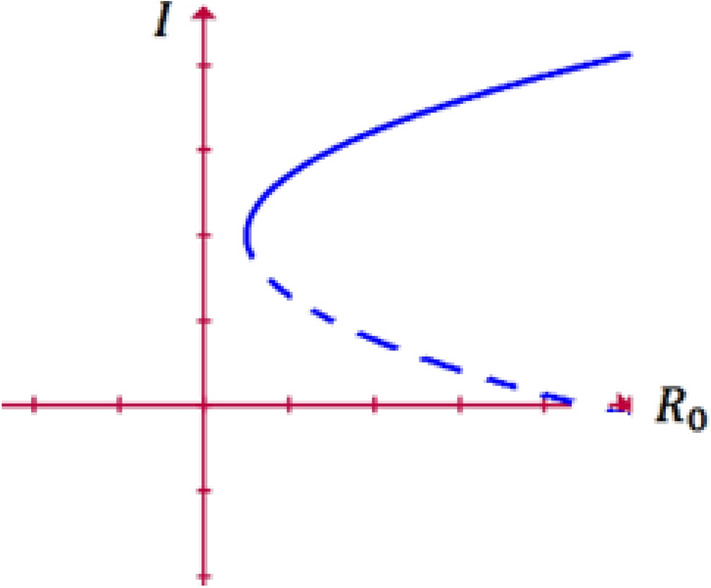
Saddle node bifurcation.

A saddle-node bifurcation, tangential bifurcation, turning point bifurcation, or fold bifurcation is a local bifurcation in the field of mathematics known as bifurcation theory. A saddle-node bifurcation is a collision and disappearance of two equilibria in dynamical systems. In autonomous systems, this occurs when the critical equilibrium has one zero eigenvalue. This phenomenon is also called fold or limit point bifurcation. The term 'saddle-node bifurcation' is most often used in reference to continuous dynamical systems. Another name is blue sky bifurcation in reference to the sudden creation of two fixed points^6^. Saddle node bifurcation is illustrated in Fig. 1.

### Trans-critical bifurcation

**Figure 2:**
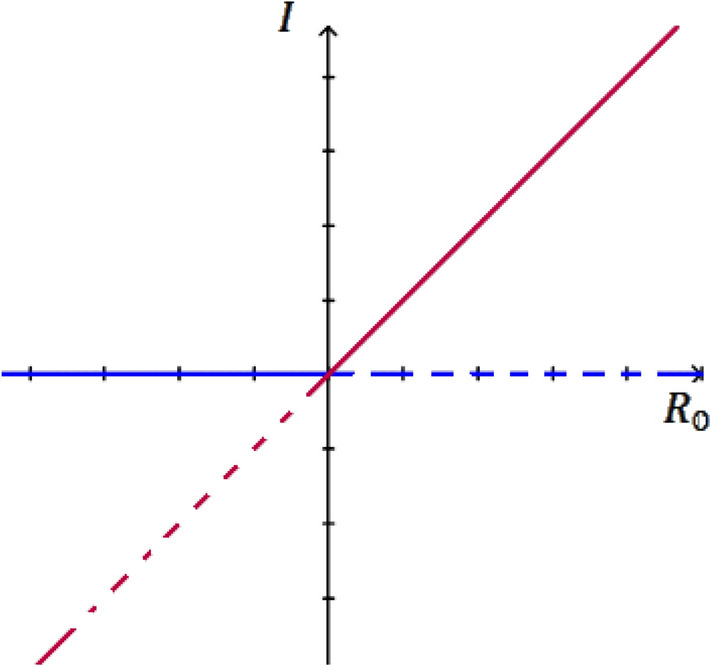
Trans-critical bifurcation.

A trans-critical bifurcation is a distinct type of local bifurcation in the mathematical field of bifurcation theory, and it is distinguished by an equilibrium whose real portion goes through zero. A trans-critical bifurcation is one in which a fixed point exists for all values of a parameter and is never destroyed. However, such a fixed point interchanges its stability with another fixed point as the parameter is varied (see Fig. 2). In other words, both before and after the bifurcation, there is one unstable and one stable fixed point. However, their stability is exchanged when they collide. So the unstable fixed point becomes stable and vice versa^5,6^.

### Pitchfork bifurcation

**Figure 3:**
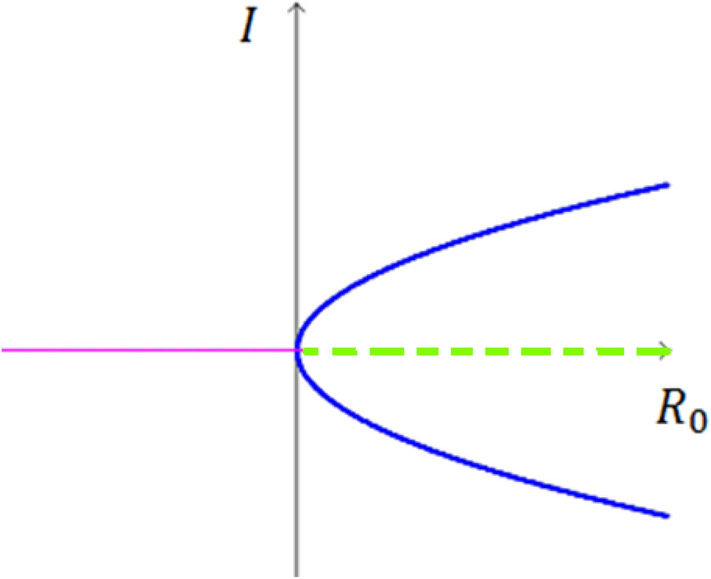
Pitchfork bifurcation.

A specific kind of local bifurcation known as a "pitchfork bifurcation" occurs when the system changes from having one fixed point to having three fixed points (it is described in Fig. 3). Pitchfork bifurcations are two types – supercritical and subcritical^11^.

### Forward bifurcation

**Figure 4:**
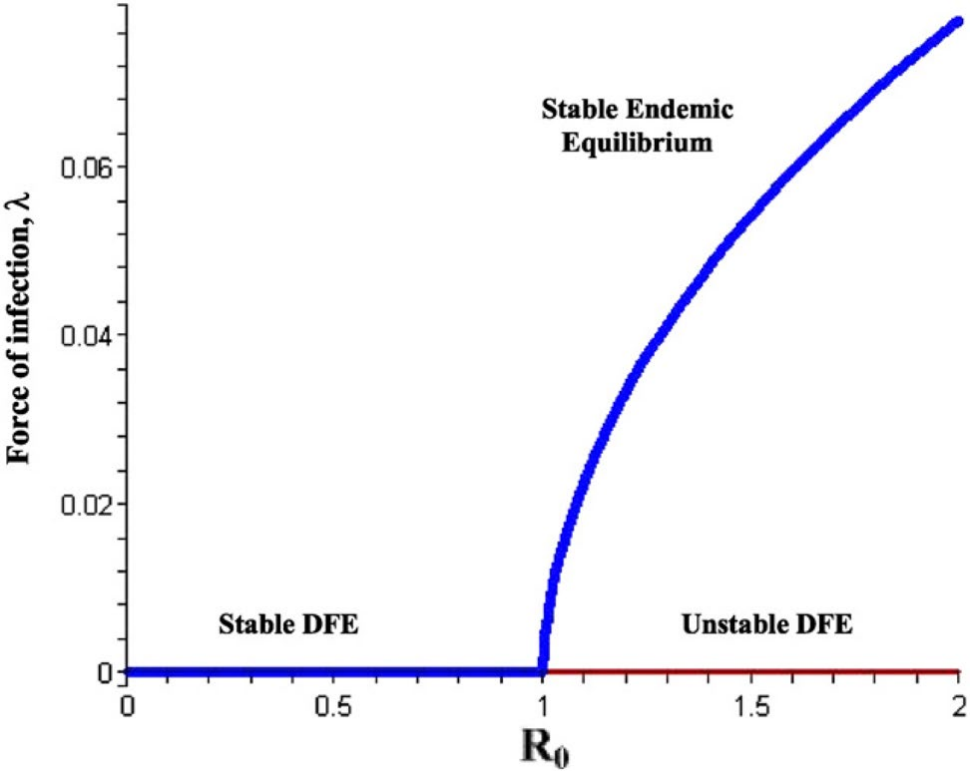
Forward bifurcation.

Epidemiologically, when reproduction number ${R}_{0}$ is less than unity, a small influx of infected individuals will not generate large outbreaks, and the disease dies out in time (in this case, the corresponding disease free equilibrium point is asymptotically-stable). On the other hand, the disease will persists if ${R}_{0}$ exceeds unity, where a stable endemic equilibrium exists. This phenomenon, illustrated in Fig. 4, where the disease-free equilibrium loses its stability and a stable endemic equilibrium appears as ${R}_{0}$ increases through one, is known as forward bifurcation^13^. Some of the main characteristics of forward bifurcation are^11^:

1. the absence of positive (endemic) equilibria near the DFE when ${R}_{0} < 1$ (in this setting, the DFE is often the only equilibrium when ${R}_{0} < 1$)
2. a low level of endemicity when ${R}_{0}$ is slightly above unity.

The forward bifurcation phenomenon has been observed in numerous disease transmission models^4,11^. For models that exhibit forward bifurcation, the requirement ${R}_{0} < 1$ is necessary and sufficient for disease elimination.

### Backward bifurcation

**Figure 5:**
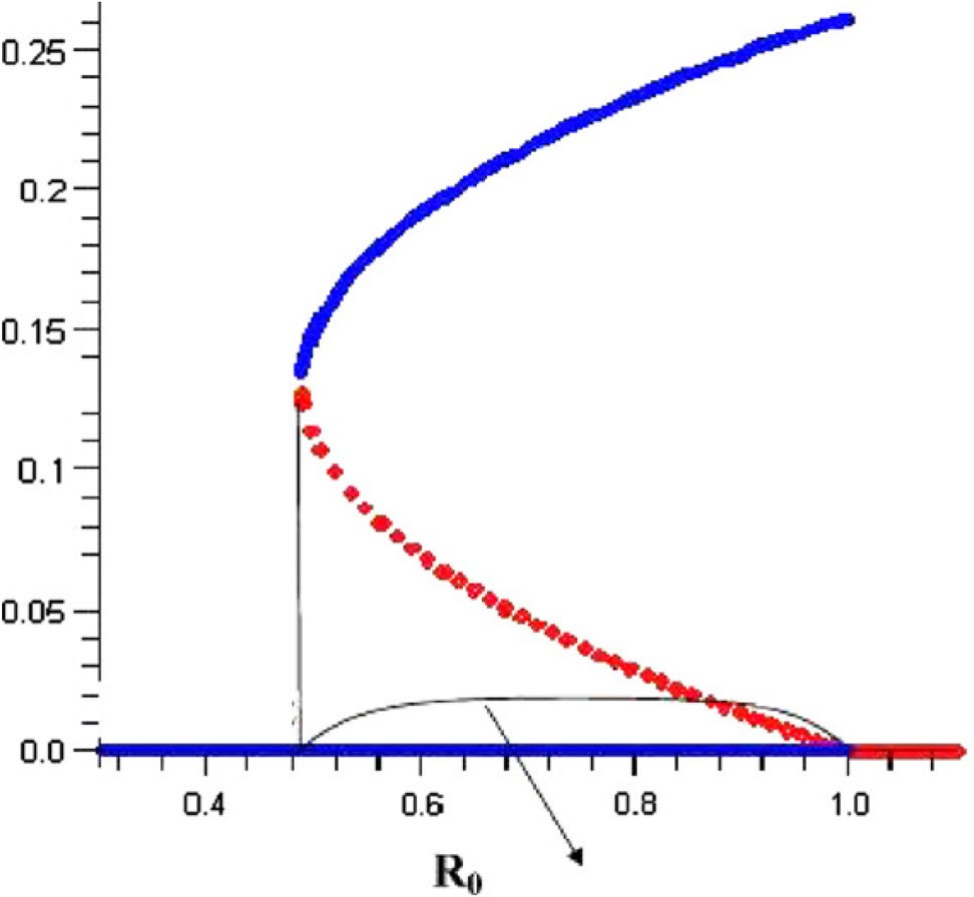
Backward bifurcation.

When forward bifurcation occurs, the condition R_0_ < 1 is usually a necessary and sufficient condition for disease eradication. Backward bifurcation is a bifurcation where the locally-asymptotically stable DFE co-exists with a locally-asymptotically endemic equilibrium when R_0_ < 1 (see Fig. 5). The epidemiological implication of backward bifurcation is that the requirement ${R}_{0} < 1$, while necessary, is not sufficient for effective disease control. In a backward bifurcation setting, once ${R}_{0}$ crosses unity, the disease can invade to a relatively high endemic level. In this case, decreasing ${R}_{0}$ to its former level will not necessarily make the disease disappear^11^.

### Methods of identifying types of bifurcation

To know the qualitative behavior of the solution of the given n-dimensional dynamical system:

1 $$ \dot{x} = \frac{dx}{{dt}} = f\left( {x,\mu } \right), x \in {\mathbb{R}}^{n} \;and\;\mu \in {\mathbb{R}}^{m} $$

near the non-hyperbolic equilibrium points, changes as the vector field $f$ passes through a point in the bifurcation set or as the parameter $\mu$ varies through a bifurcation value ${\mu }_{0}$; we use the following criteria.

#### **Theorem 1**

(Sotomayor’s theorem). Suppose that $f ({{\varvec{x}}}_{0},\boldsymbol{ }{{\varvec{\mu}}}_{0}) = 0$ and that the $n \times n$ matrix $A = Df \left({{\varvec{x}}}_{0},\boldsymbol{ }{{\varvec{\mu}}}_{0}\right)$ has a simple eigenvalue $\lambda = 0$ with eigenvector $v$ and that ${A}^{T}$ has an eigenvector $w$ corresponding to the eigenvalue $\lambda = 0$. Furthermore, suppose that A has k eigenvalues with negative real part and $\left(n - k - 1\right)$ eigenvalues with positive real part and that the following conditions are satisfied.

1. If ${w}^{T}{f}_{\mu }\left({{\varvec{x}}}_{0},{{\varvec{\mu}}}_{0}\right)\ne 0, {w}^{T}\left[{D}^{2}f\left({{\varvec{x}}}_{0},{{\varvec{\mu}}}_{0}\right)\left({\varvec{v}},{\varvec{v}}\right)\right]\ne 0,$ the system (1) experiences a saddle-node bifurcation at the equilibrium point ${{\varvec{x}}}_{0}$ as the parameter ${\varvec{\mu}}$ passes through the bifurcation value ${\varvec{\mu}}={{\varvec{\mu}}}_{0}$.
2. If ${w}^{T}{f}_{\mu }\left({{\varvec{x}}}_{0},{{\varvec{\mu}}}_{0}\right)=0$,${w}^{T}\left[Df\left({{\varvec{x}}}_{0},{{\varvec{\mu}}}_{0}\right){\varvec{v}}\right]\ne 0$ and ${w}^{T}\left[{D}^{2}f\left({{\varvec{x}}}_{0},{{\varvec{\mu}}}_{0}\right)\left({\varvec{v}},{\varvec{v}}\right)\right]\ne 0,$then system^7^ (1) experiences a trans-critical bifurcation at the equilibrium point ${{\varvec{x}}}_{0}$ as the parameter ${\varvec{\mu}}$ varies through the bifurcation value ${\varvec{\mu}}={{\varvec{\mu}}}_{0}$
3. If ${w}^{T}{f}_{\mu }\left({{\varvec{x}}}_{0},{{\varvec{\mu}}}_{0}\right)=0$,${w}^{T}[D{f}_{\mu }({{\varvec{x}}}_{0},{{\varvec{\mu}}}_{0}){\varvec{v}}] \ne 0$, ${w}^{T}\left[{D}^{2}f\left({{\varvec{x}}}_{0},{{\varvec{\mu}}}_{0}\right)\left({\varvec{v}},{\varvec{v}}\right)\right]= 0$ and ${w}^{T}[{D}^{3}f({{\varvec{x}}}_{0},{{\varvec{\mu}}}_{0})({\varvec{v}},{\varvec{v}},{\varvec{v}})] \ne 0$, then system (1) experiences a pitchfork bifurcation at the equilibrium point ${{\varvec{x}}}_{0}$ as the parameter ${\varvec{\mu}}$ varies through the bifurcation value ${\varvec{\mu}}={{\varvec{\mu}}}_{0}$.

#### **Theorem 2**

^14^. Consider the following general system of ordinary differential equations with a parameter $\mu$

2 $$ \frac{dx}{{dt}} = f\left( {{\varvec{x}},{\varvec{\mu}}} \right), f:{\mathbb{R}}^{n} \times {\mathbb{R}} \to {\mathbb{R}}\;and\;f\varepsilon {\mathbb{C}}^{2} \left( {{\mathbb{R}}^{n} \times {\mathbb{R}}} \right) $$

where **0** is an equilibrium point of the system (that is, $f\left(0,\mu \right)=0$ for all $\mu$) and assume.

${A}_{1}$: $A={D}_{x}f\left(0,0\right)=(\frac{\partial {f}_{i}}{\partial {x}_{j}}(0,0))$ is the linearization matrix of the system^8^ around the equilibrium $0$ with ${\varvec{\mu}}$ evaluated at $0$. Zero is a simple eigenvalue of $A$ and other eigenvalues of $A$ have negative real parts;

${A}_{2}$: Matrix $A$ has a right eigenvector $w$ and a left eigenvector ${\varvec{v}}$(each corresponding to the zero eigenvalue).

Let ${f}_{k}$ be the ${k}^{th}$ component of $f$ and

$$a=\sum_{k,i,j=1}^{n}{v}_{k}{w}_{i}{w}_{j}\frac{{\partial }^{2}{f}_{k}}{\partial {x}_{i}\partial {x}_{j}}(0,0)$$

$$b=\sum_{k,i,j=1}^{n}{v}_{k}{w}_{i}\frac{{\partial }^{2}{f}_{k}}{\partial {x}_{i}\partial \mu }(0,0)$$

The local dynamics of the system around $0$ is totally determined by the signs of $a$ and $b$

1. $Let\, a > 0, b > 0$. When ${\varvec{\mu}}< 0$ with $|{\varvec{\mu}}| \ll 1$, $0$ is locally asymptotically stable and there exists a positive unstable equilibrium; when $0 <{\varvec{\mu}}\ll 1$, $0$ is unstable and there exists a negative, locally asymptotically stable equilibrium;
2. $Let\, a< 0, b< 0$. When ${\varvec{\mu}}< 0$ with $|{\varvec{\mu}}| \ll 1$, $0$ is unstable; when $0 <{\varvec{\mu}}\ll 1$ , 0 is locally asymptotically stable equilibrium, and there exists a positive unstable equilibrium;
3. $Let\, a> 0, b< 0$. When $\mu < 0$ with $|{\varvec{\mu}}| \ll 1$, $0$ is unstable, and there exists a locally asymptotically stable negative equilibrium; when $0 <{\varvec{\mu}}\boldsymbol{ }\ll 1$, 0 is stable, and a positive unstable equilibrium appears;
4. $Let\, a< 0, b> 0$. When ${\varvec{\mu}}$ changes from negative to positive, 0 changes its stability from stable to unstable. Correspondingly a negative unstable equilibrium becomes positive and locally asymptotically stable.
5. When $a > 0$ and $b > 0$, the bifurcation at ${\varvec{\mu}}= 0$ is subcritical (backward bifurcation).
6. If $a< 0$ and $b > 0$, then the bifurcation at ${\varvec{\mu}}= 0$ is supercritical (forward bifurcation).

### Dynamics of hepatitis B virus in the host

Hepatitis is inflammation of the liver, usually producing swelling and, in many cases, permanent damage to liver tissues (cirrhosis). A number of agents can cause hepatitis, including infectious diseases, chemical poisons, drugs, and alcohol. Hepatitis B virus (HBV) interferes with the function of the liver by replicating in the liver cells called hepatocytes. HBV is spread through contact with infected bodily fluids such as blood, semen, and cervical fluid. Although the virus is found in every bodily secretion, it is not transferred through casual contact.

Infections of HBV occur only if the virus is able to enter the blood stream and reach the liver. Once in the liver, the virus reproduces and releases large numbers of new viruses into the blood stream^15^, HBV can be either acute or chronic stage. The acute form is a short-term illness that occurs within the first 6 months after a person is exposed to HBV. The diseases can become chronic stage when the HBV occurs more than 6 months after a person is exposed, although this does not always happen and, particularly in the case of hepatitis B, the likelihood of chronicity depends on a person’s age at the time of infection. Chronic hepatitis B infection is a silent killer. Without screening for infection, many acutely and chronically infected persons are not aware that they have been infected until symptoms of advanced liver disease appear^16^.

Hepatitis B is a potentially life-threatening liver infection caused by the hepatitis B virus. It is a major global health problem. It can cause chronic liver disease and chronic infection and puts people at high risk of death from cirrhosis of the liver and hepatocellular carcinoma (liver cancer)^17^. Infections of hepatitis B occur only if the virus is able to enter the blood stream and reach the liver. Once in the liver, the virus reproduces and releases large numbers of new viruses into the blood stream^18^.

Treatment strategies include drug therapy for HBV and liver transplantation incases of end-stage liver disease. However, these treatments are expensive and can produce significant side effects^6^. Entecavir is off-patent, but availability and costs vary widely. Tenofovir is protected by a patent until 2018 in most upper-middle- and high-income countries, where the cost ranged from US$ 400 to US$ 1,500 for a year of treatment in February 2017. Additionally, it is known that many patients with liver transplants have experienced HBV reinfection, illustrating that treatments may not result in a permanent cure. Thus, in order to analyze the within-host dynamics of HBV, mathematical modeling is introduced.

Patients with liver transplants may not be fully cured from the viral infection. Because of this, speculation arises as to how individuals can become re-infected following medical and/or surgical treatment. Viral particles, present in the blood stream, may leads to the construction of an additional compartment in the model, the blood compartment. The presence of a weak and narrowly focused cellular immune response is unable to control HBV replication, leading to viral persistence and progressive liver injury^19^.

In this paper, we consider a model which includes a logistic growth term for infected liver and blood cells, a mass action term for infection of uninfected cells, a free virus term, a loss of free viruses on infection of a cell, and a non-Cytolytic cure process with specific CD8^+^ T cells that could inhibit HBV replication.

### The mathematical model

#### Assumptions

**Figure 6:**
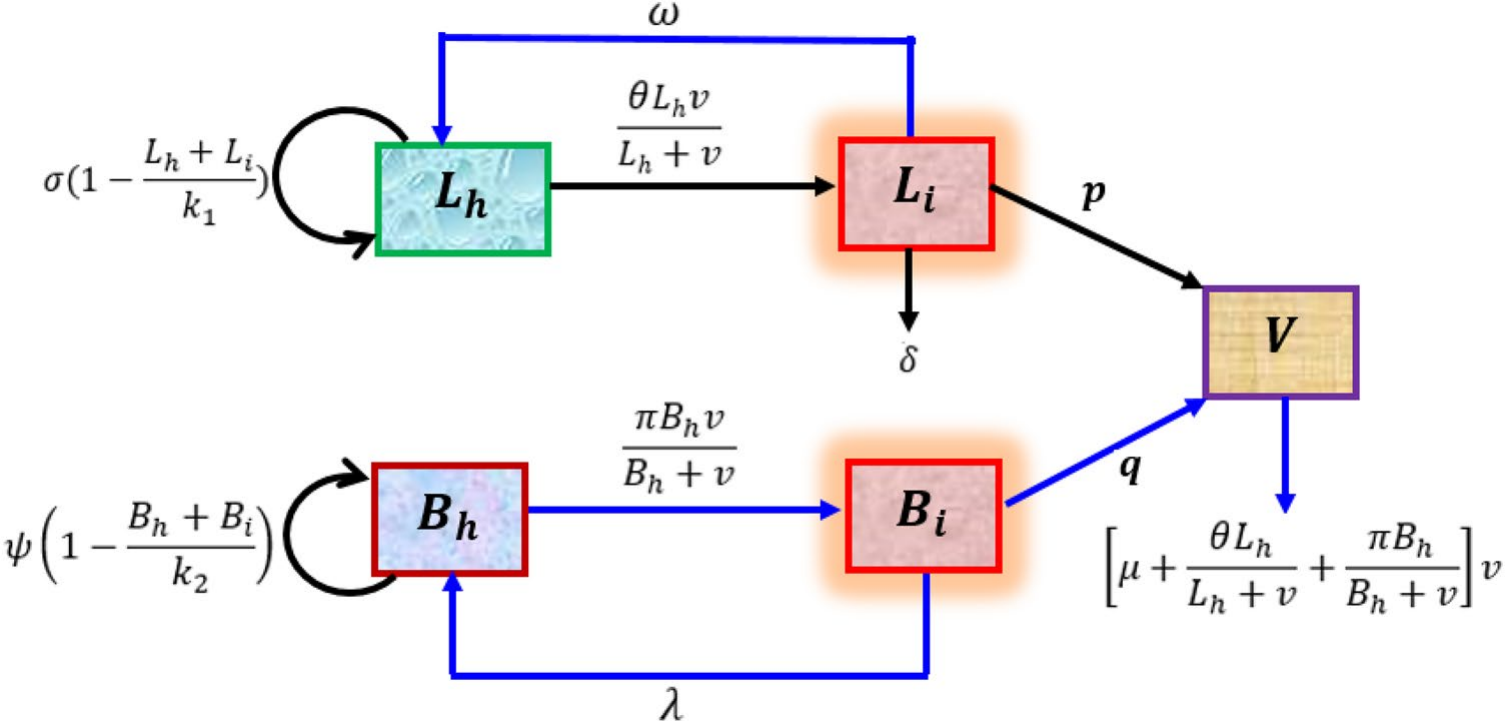
The in-host dynamics of HBV.

Let ${L}_{h}(t)$ is the number of healthy liver cell (hepatocyte), ${L}_{i}(t)$ is the number of infected liver cell, $v(t)$ is the concentration of free viruses in the liver and blood, ${B}_{h}(t)$ is the number of healthy blood cell and ${B}_{i}(t)$ is the number of infected blood cell at a time t. The epidemiological feature of the dynamics of HBV within the host (see Fig. 6) has the following assumptions:

- HBV attacks both healthy liver cell and blood cells.
- Once the liver cell and blood cell are infected; they never infected again.
- Healthy liver cell and blood cell are replicate/proliferate because of stem cell by logistic growth $\sigma \left(1-\frac{{L}_{h}+{L}_{i}}{{k}_{1}}\right)$ and $\psi \left(1-\frac{{B}_{h}+{B}_{i}}{{k}_{2}}\right)$ respectively.
- The infected hepatocytes and blood cell do not proliferate.
- Healthy liver cell and blood cell are infected by the mass action low $\frac{\theta {L}_{h}v}{{L}_{h}+v}$ and $\frac{\pi {B}_{h}v}{{B}_{h}+v}$ respectively.
- Infected liver cell and blood cell are producing free additional viruses.
- Infected cells are cured by non-Cytolytic cure processes.
- Infected cells and viruses are naturally died.
- To decrease or eliminate HBV production and viral infection in the liver $\frac{\theta {L}_{H}v}{{L}_{H}+v}+\frac{\pi {B}_{h}v}{{B}_{h}+v}$ must be reduced.

#### The flow chart of the model

The parameters in the model are defined in Table 1.

**Table 1:** Meaning of parameters in the HBV model.

Parameters	Meaning of parameters
$\sigma$	Intrinsic growth rate of healthy liver cell
$\psi$	Intrinsic growth rate of healthy blood cell
k_1_	Carrying capacity of the liver for liver cell
k_2_	Carrying capacity of the blood for blood cell
$\theta$	Rate of infection of liver cell by free virus
$\pi$	Rate of infection of blood cell by free virus
$\omega$	Rate of cure of infected liver cells by non-Cytolytic cure process
$\lambda$	Rate of cure of infected blood cells by non-Cytolytic cure process
p	Rate of release of free viruses by an infected liver cell
q	Rate of release of free viruses by an infected blood cell
$\delta$	Death rate of infected liver cells
$\eta$	Death rate of infected blood cells
$\mu$	Death rate of free virus

#### The dynamics of the model

3 $$\frac{d{L}_{h}}{dt}=\sigma \left[1-\frac{{L}_{h}+{L}_{i}}{{k}_{1}}\right]{L}_{h}+\omega {L}_{i}-\frac{\theta {L}_{h}v}{{L}_{h}+v}$$

4 $$\frac{d{L}_{i}}{dt}=\frac{\theta {L}_{h}v}{{L}_{h}+v}-\left(\delta +\omega \right){L}_{i}$$

5 $$\frac{dv}{dt}=p{L}_{i}+q{B}_{i}-\left[\mu +\frac{\theta {L}_{h}}{{L}_{h}+v}+\frac{\pi {B}_{h}}{{B}_{h}+v}\right]v$$

6 $$\frac{d{B}_{h}}{dt}=\psi \left[1-\frac{{B}_{h}+{B}_{i}}{{k}_{2}}\right]{B}_{h}+\lambda {B}_{i}-\frac{\pi {B}_{h}v}{{B}_{h}+v}$$

7 $$\frac{d{B}_{i}}{dt}=\frac{\pi {B}_{h}v}{{B}_{h}+v}-\left(\lambda +\eta \right){B}_{i}$$

In this paper, I have discussed about bifurcation analysis of hepatitis B virus with non-Cytolytic cure process (without killing cells) on infected liver and blood cells. I consider the dynamics of HBV in the host, the way how one who live with chronic HBV can prolong his life expectancy by lysis of infected liver cell and infected blood cell.

The disease free equilibrium point ${E}_{0}$ and reproduction number ${R}_{0}$ of the model are:

$$ E_{0} \left( {L_{h} ,L_{i} ,v, B_{h} , B_{i} } \right) = \left( {k_{1} ,0,0, k_{2} , 0} \right)\;and\;R_{0} = \frac{p\theta }{{\left( {\delta + \omega } \right)\left( {\mu + \pi + \theta } \right)}} + \frac{q\pi }{{\left( {\lambda + \eta } \right)\left( {\mu + \pi + \theta } \right)}}. $$

Here let $x=x({L}_{h},{L}_{i},{v, B}_{h}, {B}_{i})$. Let ${\theta }^{*}=\theta$ be a bifurcation parameter at ${R}_{0}=1;$ then,

$${R}_{0}=\frac{p\theta }{\left(\delta +\omega \right)\left(\mu +\pi +\theta \right)}+\frac{q\pi }{\left(\lambda +\eta \right)\left(\mu +\pi +\theta \right)}=\frac{p{\theta }^{*}}{\left(\delta +\omega \right)\left(\mu +\pi +{\theta }^{*}\right)}+\frac{q\pi }{\left(\lambda +\eta \right)\left(\mu +\pi +{\theta }^{*}\right)}=1$$

$\Leftrightarrow {\theta }^{*}=\frac{\frac{q\pi }{\lambda +\eta }-\left(\mu +\pi \right)}{1-\frac{p}{\delta +\omega }}$ … (*). Then the Jacobean matrix of the dynamical system of (3–7) evaluated at disease free equilibrium point ${E}_{0}=\left({k}_{1},\mathrm{0,0}, {k}_{2}, 0\right)$ is given by:

$$J({k}_{1},\mathrm{0,0}, {k}_{2}, 0)=J\left({E}_{0}\right)=\left[\begin{array}{cc}\begin{array}{c}-\sigma \\ \begin{array}{c}0\\ \begin{array}{c}0\\ \begin{array}{c}0\\ 0\end{array}\end{array}\end{array}\end{array}& \begin{array}{cc}\begin{array}{c}\omega -\sigma \\ \begin{array}{c}-(\delta +\omega )\\ \begin{array}{c}p\\ \begin{array}{c}0\\ 0\end{array}\end{array}\end{array}\end{array}& \begin{array}{cc}\begin{array}{c}\begin{array}{c}\begin{array}{c}-\theta \\ \theta \end{array}\\ -(\mu +\theta +\pi )\end{array}\\ \begin{array}{c}-\pi \\ \pi \end{array}\end{array}& \begin{array}{cc}\begin{array}{c}\begin{array}{c}\begin{array}{c}0\\ 0\end{array}\\ 0\end{array}\\ \begin{array}{c}-\psi \\ 0\end{array}\end{array}& \begin{array}{c}\begin{array}{c}\begin{array}{c}0\\ 0\end{array}\\ q\end{array}\\ \begin{array}{c}\lambda -\psi \\ -(\lambda +\eta )\end{array}\end{array}\end{array}\end{array}\end{array}\end{array}\right]$$

The Jacobean matrix $J({E}_{0})$ evaluated at ${\theta }^{*}$ i.e. ${\left.J({E}_{0})\right|}_{{\theta }^{*}}$ is given by:

$${\left.J({E}_{0})\right|}_{{\theta }^{*}}=\left[\begin{array}{cc}\begin{array}{c}-\sigma \\ \begin{array}{c}0\\ \begin{array}{c}0\\ \begin{array}{c}0\\ 0\end{array}\end{array}\end{array}\end{array}& \begin{array}{cc}\begin{array}{c}\omega -\sigma \\ \begin{array}{c}-(\delta +\omega )\\ \begin{array}{c}p\\ \begin{array}{c}0\\ 0\end{array}\end{array}\end{array}\end{array}& \begin{array}{cc}\begin{array}{c}\begin{array}{c}\begin{array}{c}-{\theta }^{*}\\ {\theta }^{*}\end{array}\\ -(\mu +{\theta }^{*}+\pi )\end{array}\\ \begin{array}{c}-\pi \\ \pi \end{array}\end{array}& \begin{array}{cc}\begin{array}{c}\begin{array}{c}\begin{array}{c}0\\ 0\end{array}\\ 0\end{array}\\ \begin{array}{c}-\psi \\ 0\end{array}\end{array}& \begin{array}{c}\begin{array}{c}\begin{array}{c}0\\ 0\end{array}\\ q\end{array}\\ \begin{array}{c}\lambda -\psi \\ -(\lambda +\eta )\end{array}\end{array}\end{array}\end{array}\end{array}\end{array}\right]$$

Let z be eigenvalue of ${\left.J({E}_{0})\right|}_{{\theta }^{*}}.$ Then, $$\left|\begin{array}{cc}\begin{array}{c}-(\sigma +z)\\ \begin{array}{c}0\\ \begin{array}{c}0\\ \begin{array}{c}0\\ 0\end{array}\end{array}\end{array}\end{array}& \begin{array}{cc}\begin{array}{c}\omega -\sigma \\ \begin{array}{c}-(\delta +\omega +z)\\ \begin{array}{c}p\\ \begin{array}{c}0\\ 0\end{array}\end{array}\end{array}\end{array}& \begin{array}{cc}\begin{array}{c}\begin{array}{c}\begin{array}{c}-{\theta }^{*}\\ {\theta }^{*}\end{array}\\ -(\mu +{\theta }^{*}+\pi +z)\end{array}\\ \begin{array}{c}-\pi \\ \pi \end{array}\end{array}& \begin{array}{cc}\begin{array}{c}\begin{array}{c}\begin{array}{c}0\\ 0\end{array}\\ 0\end{array}\\ \begin{array}{c}-(\psi +z)\\ 0\end{array}\end{array}& \begin{array}{c}\begin{array}{c}\begin{array}{c}0\\ 0\end{array}\\ q\end{array}\\ \begin{array}{c}\lambda -\psi \\ -(\lambda +\eta +z)\end{array}\end{array}\end{array}\end{array}\end{array}\end{array}\right|=0$$ is characteristic equation.

$$ \begin{aligned} & \Rightarrow \left( {\sigma + z} \right)\left( {\psi + z} \right)\left[ { - \left( {\delta + \omega + z} \right)\left( {\lambda + \eta + z} \right)\left( {\mu + \theta^{*} + \pi + z} \right) - \left[ { - p\theta^{*} \left( {\lambda + \eta + z} \right) - q\pi \left( {\delta + \omega + z} \right)} \right]} \right] = 0 \\ & \Rightarrow z_{1} = - \sigma \;or\; z_{2} = - \psi \;or \;\left( {\delta + \omega + z} \right)\left( {\lambda + \eta + z} \right)\left( {\mu + \theta^{*} + \pi + z} \right) + \left[ { - p\theta^{*} \left( {\lambda + \eta + z} \right) - q\pi \left( {\delta + \omega + z} \right)} \right] = 0 \\ & \Rightarrow  z_{1} = - \sigma \;or \;z_{2} = - \psi \;or\; z^{3} + \left( {\delta + \omega + \lambda + \eta + \mu + \theta^{*} + \pi } \right)z^{2} + [\left( {\delta + \omega } \right)\left( {\lambda + \eta } \right) + \left( {\delta + \omega } \right)\left( {\mu + \pi + \theta^{*} } \right) + \left( {\lambda + \eta } \right)\left( {\mu + \pi + \theta^{*} } \right) - p\theta^{*} - q\pi ]z \\ & \quad + \left( {\delta + \omega } \right)\left( {\lambda + \eta } \right)\left( {\mu + \pi + \theta^{*} } \right)\left[ {1 - R_{0} } \right] = 0 \\ \end{aligned} $$

Here, it is enough to show that $\left(\delta +\omega \right)\left(\lambda +\eta \right)\left(\mu +\pi +{\theta }^{*}\right)\left[1-{R}_{0}\right]=0$ for at least one of ${z}_{i}=0;i=3, 4, 5.$ Now,

$$ \begin{aligned} & \left( {\delta + \omega } \right)\left( {\lambda + \eta } \right)\left( {\mu + \pi + \theta^{*} } \right)\left[ {1 - R_{0} } \right] = \left( {\delta + \omega } \right)\left( {\lambda + \eta } \right)\left( {\mu + \pi + \theta^{*} } \right)\left[ {1 - \left( {\frac{{p\theta^{*} }}{{\left( {\delta + \omega } \right)\left( {\mu + \pi + \theta^{*} } \right)}} + \frac{q\pi }{{\left( {\lambda + \eta } \right)\left( {\mu + \pi + \theta^{*} } \right)}}} \right)} \right] \\ & \Leftrightarrow \left( {\delta + \omega } \right)\left( {\lambda + \eta } \right)\left( {\mu + \pi + \theta^{*} } \right)\left[ {1 - R_{0} } \right] = \left( {\delta + \omega } \right)\left( {\lambda + \eta } \right)\left[ {\left( {\mu + \pi + \theta^{*} } \right) - \frac{{p\theta^{*} }}{{\left( {\delta + \omega } \right)}} - \frac{q\pi }{{\left( {\lambda + \eta } \right)}} } \right] \\ & \Leftrightarrow \left( {\delta + \omega } \right)\left( {\lambda + \eta } \right)\left( {\mu + \pi + \theta^{*} } \right)\left[ {1 - R_{0} } \right] = \left( {\delta + \omega } \right)\left( {\lambda + \eta } \right)\left[ {\mu + \pi + \left( {1 - \frac{p}{\delta + \omega }} \right)\theta^{*} - \frac{q\pi }{{\left( {\lambda + \eta } \right)}} } \right] \\ \end{aligned} $$

$\Leftrightarrow \left(\delta +\omega \right)\left(\lambda +\eta \right)\left(\mu +\pi +{\theta }^{*}\right)\left[1-{R}_{0}\right]=$ $\left(\delta +\omega \right)\left(\lambda +\eta \right)\left[\mu +\pi +\left(1-\frac{p}{\delta +\omega }\right)[\frac{\frac{q\pi }{\lambda +\eta }-\left(\mu +\pi \right)}{1-\frac{p}{\delta +\omega }}]-\frac{q\pi }{\left(\lambda +\eta \right)}\right]$ by substitution of ${\theta }^{*}=\frac{\frac{q\pi }{\lambda +\eta }-\left(\mu +\pi \right)}{1-\frac{p}{\delta +\omega }}$ from (*) above.

$$ \begin{aligned} &   \Leftrightarrow   \left( {\delta + \omega } \right)\left( {\lambda + \eta } \right)\left( {\mu + \pi + \theta^{*} } \right)\left[ {1 - R_{0} } \right] = \left( {\delta + \omega } \right)\left( {\lambda + \eta } \right)\left[ {\left( {\mu + \pi } \right) + \frac{q\pi }{{\lambda + \eta }} - \left( {\mu + \pi } \right) - \frac{q\pi }{{\lambda + \eta }}} \right] \\ &   \Leftrightarrow   \left( {\delta + \omega } \right)\left( {\lambda + \eta } \right)\left( {\mu + \pi + \theta^{*} } \right)\left[ {1 - R_{0} } \right] = 0. \\ \end{aligned} $$

Thus

$$ \begin{aligned} & z_{1} = - \sigma \;or\; z_{2} = - \psi \;or\;z^{3} + \left( {\delta + \omega + \lambda + \eta + \mu + \theta^{*} + \pi } \right)z^{2} \\ & \qquad + \left[ {\left( {\delta + \omega } \right)\left( {\lambda + \eta } \right) + \left( {\delta + \omega } \right)\left( {\mu + \pi + \theta^{*} } \right) + \left( {\lambda + \eta } \right)\left( {\mu + \pi + \theta^{*} } \right) - p\theta^{*} - q\pi } \right]z = 0 \\ & \Rightarrow z_{1} = - \sigma or z_{2} = - \psi or z_{3} = 0 or z^{2} + \left( {\delta + \omega + \lambda + \eta + \mu + \theta^{*} + \pi } \right)z \\ & \qquad + \left[ {\left( {\delta + \omega } \right)\left( {\lambda + \eta } \right) + \left( {\delta + \omega } \right)\left( {\mu + \pi + \theta^{*} } \right) + \left( {\lambda + \eta } \right)\left( {\mu + \pi + \theta^{*} } \right) - p\theta^{*} - q\pi } \right] = 0 \\ \end{aligned} $$

Hence, **0** is the simple eigenvalue of ${\left.J({E}_{0})\right|}_{{\theta }^{*}}.$

##### **Theorem 3**

Assuming that ${\mathrm{R}}_{0}$ passes through the value ${\mathrm{R}}_{0}=1;$ then model (3–7) near the disease-free equilibrium ${E}_{0}\left({L}_{h},{L}_{i},{v, B}_{h}, {B}_{i}\right)=\left({k}_{1},\mathrm{0,0}, {k}_{2}, 0\right)$ has:

1. No saddle-node bifurcation;
2. A trans- critical bifurcation;
3. No pitchfork bifurcation

##### *Proof*

Let the eigenvalue of ${\left.J({E}_{0})\right|}_{{\theta }^{*}}$ is zero, and let.

$$\frac{d{L}_{h}}{dt}=\sigma \left[1-\frac{{L}_{h}+{L}_{i}}{{k}_{1}}\right]{L}_{h}+\omega {L}_{i}-\frac{\theta {L}_{h}v}{{L}_{h}+v}={f}_{1}$$

$$\frac{d{L}_{i}}{dt}=\frac{\theta {L}_{h}v}{{L}_{h}+v}-\left(\delta +\omega \right){L}_{i}={f}_{2}$$

$$\frac{dv}{dt}=p{L}_{i}+q{B}_{i}-\left[\mu +\frac{\theta {L}_{h}}{{L}_{h}+v}+\frac{\pi {B}_{h}}{{B}_{h}+v}\right]v={f}_{3}$$

$$\frac{d{B}_{h}}{dt}=\psi \left[1-\frac{{B}_{h}+{B}_{i}}{{k}_{2}}\right]{B}_{h}+\lambda {B}_{i}-\frac{\pi {B}_{h}v}{{B}_{h}+v}={f}_{4}$$

$$\frac{d{B}_{i}}{dt}=\frac{\pi {B}_{h}v}{{B}_{h}+v}-\left(\lambda +\eta \right){B}_{i}={f}_{5}$$

Let ${{x}_{0}=E}_{0}\left({L}_{h},{L}_{i},{v, B}_{h}, {B}_{i}\right)=\left({k}_{1},\mathrm{0,0}, {k}_{2}, 0\right)$ and ${\mu }_{0}={\theta }^{*}=\frac{\frac{q\pi }{\lambda +\eta }-\left(\mu +\pi \right)}{1-\frac{p}{\delta +\omega }}$. Then,

$$Df\left(x,\mu \right)=\left[\begin{array}{ccccc}{a}_{1}& {a}_{2}& {a}_{3}& 0& 0\\ {b}_{1}& {b}_{2}& {b}_{3}& 0& 0\\ {c}_{1}& p& {c}_{2}& {c}_{3}& q\\ 0& 0& {d}_{1}& {d}_{2}& {d}_{3}\\ 0& 0& {e}_{1}& {e}_{2}& {e}_{3}\end{array}\right],$$

where

$$ \begin{aligned} & {a}_{1}=\frac{\sigma }{{k}_{1}}\left[{k}_{1}-2{L}_{h}-{L}_{i}\right]-\frac{{\theta }^{*}{v}^{2}}{{\left({L}_{h}+v\right)}^{2}}, {a}_{2}=-\frac{\sigma }{{k}_{1}}{L}_{h}+\omega , {a}_{3}=-\frac{{\theta }^{*}{{L}_{h}}^{2}}{{\left({L}_{h}+v\right)}^{2}}, {b}_{1}=\frac{{\theta }^{*}{v}^{2}}{{\left({L}_{h}+v\right)}^{2}},\\& {b}_{2}=-\left(\delta +\omega \right), {b}_{3}=\frac{{\theta }^{*}{{L}_{h}}^{2}}{{\left({L}_{h}+v\right)}^{2}}, {c}_{1}=\frac{-{\theta }^{*}{v}^{2}}{{\left({L}_{h}+v\right)}^{2}}, {c}_{2}=-\left(\mu +\frac{{\theta }^{*}{{L}_{h}}^{2}}{{\left({L}_{h}+v\right)}^{2}}+\frac{\pi {{B}_{h}}^{2}}{{\left({B}_{h}+v\right)}^{2}}\right), \\ &{c}_{3}=\frac{-\pi {v}^{2}}{{\left({B}_{h}+v\right)}^{2}}, {d}_{1}=-\frac{\pi {{B}_{h}}^{2}}{{\left({B}_{h}+v\right)}^{2}}, {d}_{2}=\frac{\psi }{{k}_{2}}\left[{k}_{2}-2{B}_{h}-{B}_{i}\right]-\frac{\pi {v}^{2}}{{\left({B}_{h}+v\right)}^{2}}, {d}_{3}=-\frac{\psi }{{k}_{2}}{B}_{h}+\lambda , \\ &{e}_{1}=\frac{\pi {{B}_{h}}^{2}}{{\left({B}_{h}+v\right)}^{2}}, {e}_{2}=\frac{\pi {v}^{2}}{{\left({B}_{h}+v\right)}^{2}}, {e}_{3}=-\left(\lambda +\eta \right)\end{aligned} $$

$$\Rightarrow Df\left({x}_{0},{\mu }_{0}\right)=\left[\begin{array}{cc}\begin{array}{c}-\sigma \\ \begin{array}{c}0\\ \begin{array}{c}0\\ \begin{array}{c}0\\ 0\end{array}\end{array}\end{array}\end{array}& \begin{array}{cc}\begin{array}{c}\omega -\sigma \\ \begin{array}{c}-\left(\delta +\omega \right)\\ \begin{array}{c}p\\ \begin{array}{c}0\\ 0\end{array}\end{array}\end{array}\end{array}& \begin{array}{cc}\begin{array}{c}\begin{array}{c}\begin{array}{c}-{\theta }^{*}\\ {\theta }^{*}\end{array}\\ -\left(\mu +{\theta }^{*}+\pi \right)\end{array}\\ \begin{array}{c}-\pi \\ \pi \end{array}\end{array}& \begin{array}{cc}\begin{array}{c}\begin{array}{c}\begin{array}{c}0\\ 0\end{array}\\ 0\end{array}\\ \begin{array}{c}-\psi \\ 0\end{array}\end{array}& \begin{array}{c}\begin{array}{c}\begin{array}{c}0\\ 0\end{array}\\ q\end{array}\\ \begin{array}{c}\lambda -\psi \\ -\left(\lambda +\eta \right)\end{array}\end{array}\end{array}\end{array}\end{array}\end{array}\right]=A.$$

$${D}^{2}f\left({x}_{0},{\mu }_{0}\right)=\left[\begin{array}{ccccccccccccccccccccccccc}-\frac{2\sigma }{{k}_{1}}& -\frac{\sigma }{{k}_{1}}& 0& 0& 0& -\frac{\sigma }{{k}_{1}}& 0& 0& 0& 0& 0& 0& \frac{2{\theta }^{*}}{{k}_{1}}& 0& 0& 0& 0& 0& 0& 0& 0& 0& 0& 0& 0\\ 0& 0& 0& 0& 0& 0& 0& 0& 0& 0& 0& 0& -\frac{2{\theta }^{*}}{{k}_{1}}& 0& 0& 0& 0& 0& 0& 0& 0& 0& 0& 0& 0\\ 0& 0& 0& 0& 0& 0& 0& 0& 0& 0& 0& 0& 2(\frac{\theta }{{k}_{1}}+\frac{\pi }{{k}_{2}})& 0& 0& 0& 0& 0& 0& 0& 0& 0& 0& 0& 0\\ 0& 0& 0& 0& 0& 0& 0& 0& 0& 0& 0& 0& \frac{2\pi }{{k}_{2}}& 0& 0& 0& 0& 0& -\frac{2\psi }{{k}_{2}}& -\frac{\psi }{{k}_{2}}& 0& 0& 0& -\frac{\psi }{{k}_{2}}& 0\\ 0& 0& 0& 0& 0& 0& 0& 0& 0& 0& 0& 0& -\frac{2\pi }{{k}_{2}}& 0& 0& 0& 0& 0& 0& 0& 0& 0& 0& 0& 0\end{array}\right]$$

$$\Rightarrow AV=0, Where\, V={\left({v}_{1}, {v}_{2}, {v}_{3}, {v}_{4}, {v}_{5}\right)}^{T}\,and\, 0={\left(0 0, 0, 0, 0, 0\right)}^{T}$$

$$\Rightarrow \left[\begin{array}{cc}\begin{array}{c}-\sigma \\ \begin{array}{c}0\\ \begin{array}{c}0\\ \begin{array}{c}0\\ 0\end{array}\end{array}\end{array}\end{array}& \begin{array}{cc}\begin{array}{c}\omega -\sigma \\ \begin{array}{c}-\left(\delta +\omega \right)\\ \begin{array}{c}p\\ \begin{array}{c}0\\ 0\end{array}\end{array}\end{array}\end{array}& \begin{array}{cc}\begin{array}{c}\begin{array}{c}\begin{array}{c}-{\theta }^{*}\\ {\theta }^{*}\end{array}\\ -\left(\mu +{\theta }^{*}+\pi \right)\end{array}\\ \begin{array}{c}-\pi \\ \pi \end{array}\end{array}& \begin{array}{cc}\begin{array}{c}\begin{array}{c}\begin{array}{c}0\\ 0\end{array}\\ 0\end{array}\\ \begin{array}{c}-\psi \\ 0\end{array}\end{array}& \begin{array}{c}\begin{array}{c}\begin{array}{c}0\\ 0\end{array}\\ q\end{array}\\ \begin{array}{c}\lambda -\psi \\ -\left(\lambda +\eta \right)\end{array}\end{array}\end{array}\end{array}\end{array}\end{array}\right]\left[\begin{array}{c}{v}_{1}\\ {v}_{2}\\ \begin{array}{c}{v}_{3}\\ {v}_{4}\\ {v}_{5}\end{array}\end{array}\right]=\left[\begin{array}{c}0\\ 0\\ \begin{array}{c}0\\ 0\\ 0\end{array}\end{array}\right]$$

$$\Rightarrow \left\{\begin{array}{c}-\sigma {v}_{1}+\left(\omega -\sigma \right){v}_{2}-{\theta }^{*}{v}_{3}=0\dots (a)\\ -\left(\delta +\omega \right){v}_{2}+{\theta }^{*}{v}_{3}=0\dots .(b)\\ \begin{array}{c}p{v}_{2}-\left(\mu +{\theta }^{*}+\pi \right){v}_{3}+q{v}_{5}=0\dots (c)\\ -\pi {v}_{3}-\psi {v}_{4}+\left(\lambda -\psi \right){v}_{5}=0\dots (d)\\ \pi {v}_{3}-\left(\lambda +\eta \right){v}_{5}=0\dots (e)\end{array}\end{array}\right.$$

From (b), we have ${v}_{3}=\frac{\delta +\omega }{{\theta }^{*}}{v}_{2}.$ Substitution of ${v}_{3}$ in (a), provides:

$$-\sigma {v}_{1}+\left(\omega -\sigma \right){v}_{2}-{\theta }^{*}\left(\frac{\delta +\omega }{{\theta }^{*}}\right){v}_{2}=0$$

$\Rightarrow {v}_{1}=-\frac{\left(\sigma +\delta \right)}{\sigma }{v}_{2} .$ From (e) and (c), we have ${v}_{5}=\frac{\pi }{\lambda +\eta }{v}_{3}=\frac{\pi \left(\delta +\omega \right)}{{\theta }^{*}(\lambda +\eta )}{v}_{2}=\frac{\left[\frac{\left(\mu +{\theta }^{*}+\pi \right)\left(\delta +\omega \right)}{{\theta }^{*}}-p\right]}{q}{v}_{2}$.

From (d) we have ${v}_{4}=-\frac{\pi \left(\delta +\omega \right)\left(\psi +\eta \right)}{{\theta }^{*}\psi \left(\lambda +\eta \right)}{v}_{2}$. Let ${v}_{2}=1$

Thus, $$V={\left(-\frac{\left(\sigma +\delta \right)}{\sigma }, 1, \frac{\delta +\omega }{{\theta }^{*}},-\frac{\pi \left(\delta +\omega \right)\left(\psi +\eta \right)}{{\theta }^{*}\psi \left(\lambda +\eta \right)}, \frac{\pi \left(\delta +\omega \right)}{{\theta }^{*}\left(\lambda +\eta \right)}\right)}^{T},$$

$$\left(V,V\right)=\left(\left[\begin{array}{c}-\frac{\left(\sigma +\delta \right)}{\sigma }\\ 1\\ \begin{array}{c}\frac{\delta +\omega }{{\theta }^{*}}\\ -\frac{\pi \left(\delta +\omega \right)\left(\psi +\eta \right)}{{\theta }^{*}\psi \left(\lambda +\eta \right)}\\ \frac{\pi \left(\delta +\omega \right)}{{\theta }^{*}\left(\lambda +\eta \right)}\end{array}\end{array}\right], \left[\begin{array}{c}-\frac{\left(\sigma +\delta \right)}{\sigma }\\ 1\\ \begin{array}{c}\frac{\delta +\omega }{{\theta }^{*}}\\ -\frac{\pi \left(\delta +\omega \right)\left(\psi +\eta \right)}{{\theta }^{*}\psi \left(\lambda +\eta \right)}\\ \frac{\pi \left(\delta +\omega \right)}{{\theta }^{*}\left(\lambda +\eta \right)}\end{array}\end{array}\right]\right)={\left(\begin{array}{c}{a}_{1}\\ {a}_{2}\\ {a}_{3}\\ \vdots \\ {a}_{25}\end{array}\right)}_{25\times 1}$$

where:

$$ \begin{aligned} & {a}_{1}={\left[\frac{\left(\sigma +\delta \right)}{\sigma }\right]}^{2}, {a}_{2}=-\frac{\left(\sigma +\delta \right)}{\sigma }, {a}_{3}=-\frac{\left(\sigma +\delta \right)\left(\delta +\omega \right)}{\sigma {\theta }^{*}}, {a}_{4}=\frac{\pi \left(\delta +\omega \right)\left(\psi +\eta \right)\left(\sigma +\delta \right)}{\sigma \psi {\theta }^{*}\left(\lambda +\eta \right)}, \\ &{a}_{5}=-\frac{\pi \left(\sigma +\delta \right)\left(\delta +\omega \right)}{\sigma {\theta }^{*}\left(\lambda +\eta \right)}, {a}_{6}=-\frac{\sigma +\delta }{\sigma }, {a}_{7}=1,\end{aligned} $$

$${a}_{8}=\frac{\delta +\omega }{{\theta }^{*}}, {a}_{9}=-\frac{\pi \left(\delta +\omega \right)\left(\psi +\eta \right)}{{\theta }^{*}\psi \left(\lambda +\eta \right)}, {a}_{10}=\frac{\pi \left(\delta +\omega \right)}{{\theta }^{*}\left(\lambda +\eta \right)}, {a}_{11}=-\frac{\left(\sigma +\delta \right)\left(\delta +\omega \right)}{\sigma {\theta }^{*}}, {a}_{12}=\frac{\delta +\omega }{{\theta }^{*}}, {a}_{13}={\left(\frac{\delta +\omega }{{\theta }^{*}}\right)}^{2},$$

$$ \begin{aligned} & {a}_{14}=-\frac{\pi {\left(\delta +\omega \right)}^{2}\left(\psi +\eta \right)}{{{\theta }^{*}}^{2}\psi \left(\lambda +\eta \right)}, {a}_{15}=\frac{\pi {\left(\delta +\omega \right)}^{2}}{{{\theta }^{*}}^{2}\left(\lambda +\eta \right)}, {a}_{16}=\frac{\pi \left(\sigma +\delta \right)\left(\delta +\omega \right)\left(\psi +\eta \right)}{\sigma \psi {\theta }^{*}\left(\lambda +\eta \right)},\\ & {a}_{17}=-\frac{\pi \left(\delta +\omega \right)\left(\psi +\eta \right)}{{\theta }^{*}\psi \left(\lambda +\eta \right)}, {a}_{18}=-\frac{\pi {\left(\delta +\omega \right)}^{2}\left(\psi +\eta \right)}{{{\theta }^{*}}^{2}\psi \left(\lambda +\eta \right)},\end{aligned} $$

$$ \begin{aligned} & {a}_{19}={\left[\frac{\pi \left(\delta +\omega \right)\left(\psi +\eta \right)}{{\theta }^{*}\psi \left(\lambda +\eta \right)}\right]}^{2}, {a}_{20}=\frac{{-\pi }^{2}{\left(\delta +\omega \right)}^{2}\left(\psi +\eta \right)}{\psi {{\theta }^{*}}^{2}{\left(\lambda +\eta \right)}^{2}}, {a}_{21}=-\frac{\pi \left(\sigma +\delta \right)\left(\delta +\omega \right)}{\sigma {\theta }^{*}(\lambda +\eta )},\\ & {a}_{22}=\frac{\pi \left(\delta +\omega \right)}{{\theta }^{*}\left(\lambda +\eta \right)}, {a}_{23}=\frac{\pi {\left(\delta +\omega \right)}^{2}}{{{\theta }^{*}}^{2}(\lambda +\eta )},\end{aligned} $$

$${a}_{24}=-\frac{{\pi }^{2}{\left(\delta +\omega \right)}^{2}\left(\psi +\eta \right)}{\psi {{\theta }^{*}}^{2}{\left(\lambda +\eta \right)}^{2}}, {a}_{25}={\left[\frac{\pi \left(\delta +\omega \right)}{{\theta }^{*}\left(\lambda +\eta \right)}\right]}^{2}$$.

Now,

$${A}^{T}=\left[\begin{array}{ccccc}-\sigma & 0& 0& 0& 0\\ \omega -\sigma & -(\delta +\omega )& p& 0& 0\\ -{\theta }^{*}& {\theta }^{*}& -(\mu +\pi +{\theta }^{*})& -\pi & \pi \\ 0& 0& 0& -\psi & 0\\ 0& 0& q& \lambda -\psi & -(\lambda +\eta )\end{array}\right]$$

Let ${A}^{T}$ has an eigenvector $W={\left({w}_{1}, {w}_{2}, {w}_{3}, {w}_{4}, {w}_{5}\right)}^{T}$ corresponding to eigenvalue zero.

$$\Rightarrow \left[\begin{array}{ccccc}-\sigma & 0& 0& 0& 0\\ \omega -\sigma & -(\delta +\omega )& p& 0& 0\\ -{\theta }^{*}& {\theta }^{*}& -(\mu +\pi +{\theta }^{*})& -\pi & \pi \\ 0& 0& 0& -\psi & 0\\ 0& 0& q& \lambda -\psi & -(\lambda +\eta )\end{array}\right] \left[\begin{array}{c}{w}_{1}\\ {w}_{2}\\ \begin{array}{c}{w}_{3}\\ {w}_{4}\\ {w}_{5}\end{array}\end{array}\right]=\left[\begin{array}{c}0\\ 0\\ \begin{array}{c}0\\ 0\\ 0\end{array}\end{array}\right]$$

$$\Rightarrow \left\{-{\theta }^{*}{w}_{1}+\begin{array}{c}{\sigma w}_{1}=0\dots (a)\\ \left(\omega -\sigma \right){w}_{1}-\left(\delta +\omega \right){w}_{2}+p{w}_{3}=0\dots (b)\\ \begin{array}{c}{\theta }^{*}{w}_{2}-\left(\mu +\pi +{\theta }^{*}\right){w}_{3}-\pi {w}_{4}+\pi {w}_{5}=0\dots (c)\\ -\psi {w}_{4}=0\dots (d)\\ q{w}_{3}+\left(\lambda -\psi \right){w}_{4}-\left(\lambda +\eta \right){w}_{5}=0\dots (e)\end{array}\end{array}\right.$$

From (a) and (d), we have ${w}_{1}={w}_{4}=0.$ Then, from (b), ${w}_{3}=\frac{\delta +\omega }{p}{w}_{2}$ and from (c) and (e), ${w}_{5}=\frac{q\left(\delta +\omega \right)}{p\left(\lambda +\eta \right)}{w}_{2}=\frac{\left[\frac{\left(\mu +{\theta }^{*}+\pi \right)\left(\delta +\omega \right)}{p}-{\theta }^{*}\right]}{\pi }$. Then $W={\left(0, {w}_{2}, \frac{\delta +\omega }{p}{w}_{2}, 0, \frac{q\left(\delta +\omega \right)}{p\left(\lambda +\eta \right)}{w}_{2}\right)}^{T}$, $${f}_{\mu }\left(x,\mu \right)=\left[\begin{array}{c}-\frac{{L}_{h}v}{{L}_{h}+v}\\ \frac{{L}_{h}v}{{L}_{h}+v}\\ \begin{array}{c}-\frac{{L}_{h}v}{{L}_{h}+v}\\ 0\\ 0\end{array}\end{array}\right]$$ and $${f}_{\mu }\left({x}_{0},{\mu }_{0}\right)=\left[\begin{array}{c}0\\ 0\\ \begin{array}{c}0\\ 0\\ 0\end{array}\end{array}\right]$$

$$ Df_{\mu } \left( {x,\mu } \right) = \left[ {\begin{array}{*{20}c}    { - \left[ {\frac{{L_{h}  + v - 1}}{{L_{h}  + v}}} \right]v} & 0 & { - \left[ {\frac{{L_{h} }}{{L_{h}  + v}}} \right]^{2} } & 0 & 0  \\    {\left[ {\frac{{L_{h}  + v - 1}}{{L_{h}  + v}}} \right]v} & 0 & {\left[ {\frac{{L_{h} }}{{L_{h}  + v}}} \right]^{2} } & 0 & 0  \\    {\left[ {\frac{{L_{h}  + v - 1}}{{L_{h}  + v}}} \right]v} & 0 & {\left[ {\frac{{L_{h} }}{{L_{h}  + v}}} \right]^{2} } & 0 & 0  \\    0 & 0 & 0 & 0 & 0  \\    0 & 0 & 0 & 0 & 0  \\   \end{array} } \right]Df_{\mu } \left( {x_{0} ,\mu _{0} } \right) = \left[ {\begin{array}{*{20}c}    0 & 0 & { - 1} & 0 & 0  \\    0 & 0 & 1 & 0 & 0  \\    0 & 0 & 1 & 0 & 0  \\    0 & 0 & 0 & 0 & 0  \\    0 & 0 & 0 & 0 & 0  \\   \end{array} } \right]$$

**Case 1:** a)

$$ W^{T} f_{\mu } \left( {x_{0} , \mu_{0} } \right) = \left( {0, w_{2} , \frac{\delta + \omega }{p}w_{2} , 0, \frac{{q\left( {\delta + \omega } \right)}}{{p\left( {\lambda + \eta } \right)}}w_{2} } \right) \cdot \left[ {\begin{array}{*{20}c} 0 \\ 0 \\ {\begin{array}{*{20}c} 0 \\ 0 \\ 0 \\ \end{array} } \\ \end{array} } \right]{ } = 0 $$

b)

$${w}^{T}\left[{D}^{2}f\left({x}_{0},{\mu }_{0}\right)\left(v,v\right)\right]=\left(0, {w}_{2}, \frac{\delta +\omega }{p}{w}_{2}, 0, \frac{q\left(\delta +\omega \right)}{p\left(\lambda +\eta \right)}{w}_{2}\right) [\left(\begin{array}{c}\frac{-2[\delta {\vartheta }^{*}\left(\sigma -\delta \right)-\sigma {\left(\delta +\omega \right)}^{2}}{\sigma {\theta }^{*}{k}_{1}}\\ -\frac{2{\left(\delta +\omega \right)}^{2}}{{\theta }^{*}{k}_{1}}\\ \begin{array}{c}\frac{2(\pi {k}_{1}+{\theta }^{*}{k}_{2}){\left(\delta +\omega \right)}^{2}}{{{\theta }^{*}}^{2}{k}_{1}{k}_{2}}\\ \frac{2\pi {\left(\delta +\omega \right)}^{2}[\psi {\left(\lambda +\eta \right)}^{2}-\pi {\left(\psi +\eta \right)}^{2}+\psi (\lambda +\eta )]}{{k}_{2}\psi {{\theta }^{*}}^{2}{\left(\lambda +\eta \right)}^{2}}\\ -\frac{2\pi {\left(\delta +\omega \right)}^{2}}{{k}_{2}{{\theta }^{*}}^{2}}\end{array}\end{array}\right)]$$

$$\Rightarrow {w}^{T}\left[{D}^{2}f\left({x}_{0},{\mu }_{0}\right)\left(v,v\right)\right]=\frac{2{\left(\delta +\omega \right)}^{2}[{\theta }^{*}\left(\pi {k}_{1}+{\theta }^{*}{k}_{2}\right)\left(\delta +\omega \right)-p{k}_{2}{\theta }^{*}\left(\lambda +\eta \right)-{k}_{1}\pi (\lambda +\eta )(\delta +\omega )]}{p{k}_{1}{k}_{2}{{\theta }^{*}}^{2}(\lambda +\eta )}\ne 0$$

By Sotomayor theorem, this shows that the model (3–7) do not exhibit saddle-node bifurcation.

**Case 2:** a)

$${W}^{T}{f}_{\mu }\left({x}_{0}, {\mu }_{0}\right)=\left(0, {w}_{2}, \frac{\delta +\omega }{p}{w}_{2}, 0, \frac{q\left(\delta +\omega \right)}{p\left(\lambda +\eta \right)}{w}_{2}\right) \left[\begin{array}{c}0\\ 0\\ \begin{array}{c}0\\ 0\\ 0\end{array}\end{array}\right] =0$$

b)

$${W}^{T}{[Df}_{\mu }\left({x}_{0}, {\mu }_{0}\right)V]=\left(0, {w}_{2}, \frac{\delta +\omega }{p}{w}_{2}, 0, \frac{q\left(\delta +\omega \right)}{p\left(\lambda +\eta \right)}{w}_{2}\right) [\left[\begin{array}{ccccc}0& 0& -1& 0& 0\\ 0& 0& 1& 0& 0\\ 0& 0& 1& 0& 0\\ 0& 0& 0& 0& 0\\ 0& 0& 0& 0& 0\end{array}\right]\left[\begin{array}{c}-\frac{\left(\sigma +\delta \right)}{\sigma }\\ 1\\ \begin{array}{c}\frac{\delta +\omega }{{\theta }^{*}}\\ -\frac{\pi \left(\delta +\omega \right)\left(\psi +\eta \right)}{{\theta }^{*}\psi \left(\lambda +\eta \right)}\\ \frac{\pi \left(\delta +\omega \right)}{{\theta }^{*}\left(\lambda +\eta \right)}\end{array}\end{array}\right]]$$

$$\Rightarrow {W}^{T}{[Df}_{\mu }\left({x}_{0}, {\mu }_{0}\right)V]=\frac{\delta +\omega }{{\theta }^{*}}(1+\frac{\delta +\omega }{p})\ne 0$$

c)

$${w}^{T}\left[{D}^{2}f\left({x}_{0},{\mu }_{0}\right)\left(v,v\right)\right]=\left(0, 1, \frac{\delta +\omega }{p}, 0, \frac{q\left(\delta +\omega \right)}{p\left(\lambda +\eta \right)}\right)[\left(\begin{array}{c}\frac{-2[\delta {\vartheta }^{*}\left(\sigma -\delta \right)-\sigma {\left(\delta +\omega \right)}^{2}}{\sigma {\theta }^{*}{k}_{1}}\\ -\frac{2{\left(\delta +\omega \right)}^{2}}{{\theta }^{*}{k}_{1}}\\ \begin{array}{c}\frac{2(\pi {k}_{1}+{\theta }^{*}{k}_{2}){\left(\delta +\omega \right)}^{2}}{{{\theta }^{*}}^{2}{k}_{1}{k}_{2}}\\ \frac{2\pi {\left(\delta +\omega \right)}^{2}[\psi {\left(\lambda +\eta \right)}^{2}-\pi {\left(\psi +\eta \right)}^{2}+\psi (\lambda +\eta )]}{{k}_{2}\psi {{\theta }^{*}}^{2}{\left(\lambda +\eta \right)}^{2}}\\ -\frac{2\pi {\left(\delta +\omega \right)}^{2}}{{k}_{2}{{\theta }^{*}}^{2}}\end{array}\end{array}\right)]$$

$$\Rightarrow {w}^{T}\left[{D}^{2}f\left({x}_{0},{\mu }_{0}\right)\left(v,v\right)\right]=\frac{2{\left(\delta +\omega \right)}^{2}[{\theta }^{*}\left(\pi {k}_{1}+{\theta }^{*}{k}_{2}\right)\left(\delta +\omega \right)-p{k}_{2}{\theta }^{*}\left(\lambda +\eta \right)-{k}_{1}\pi (\lambda +\eta )(\delta +\omega )]}{p{k}_{1}{k}_{2}{{\theta }^{*}}^{2}(\lambda +\eta )}\ne 0$$

By Sotomayor theorem, this shows that model (3–7) exhibit trans-critical bifurcation.

**Case 3:** a)

$${W}^{T}{f}_{\mu }\left({x}_{0}, {\mu }_{0}\right)=\left(0, {w}_{2}, \frac{\delta +\omega }{p}{w}_{2}, 0, \frac{q\left(\delta +\omega \right)}{p\left(\lambda +\eta \right)}{w}_{2}\right)\left[\begin{array}{c}0\\ 0\\ \begin{array}{c}0\\ 0\\ 0\end{array}\end{array}\right] =0$$

b)

$${W}^{T}{[Df}_{\mu }\left({x}_{0}, {\mu }_{0}\right)V]=\left(0, {w}_{2}, \frac{\delta +\omega }{p}{w}_{2}, 0, \frac{q\left(\delta +\omega \right)}{p\left(\lambda +\eta \right)}{w}_{2}\right) [\left[\begin{array}{ccccc}0& 0& -1& 0& 0\\ 0& 0& 1& 0& 0\\ 0& 0& 1& 0& 0\\ 0& 0& 0& 0& 0\\ 0& 0& 0& 0& 0\end{array}\right]\left[\begin{array}{c}-\frac{\left(\sigma +\delta \right)}{\sigma }\\ 1\\ \begin{array}{c}\frac{\delta +\omega }{{\theta }^{*}}\\ -\frac{\pi \left(\delta +\omega \right)\left(\psi +\eta \right)}{{\theta }^{*}\psi \left(\lambda +\eta \right)}\\ \frac{\pi \left(\delta +\omega \right)}{{\theta }^{*}\left(\lambda +\eta \right)}\end{array}\end{array}\right]]$$

$$\Rightarrow {W}^{T}{[Df}_{\mu }\left({x}_{0}, {\mu }_{0}\right)V]=\frac{\delta +\omega }{{\theta }^{*}}(1+\frac{\delta +\omega }{p})\ne 0$$

c)

$${w}^{T}\left[{D}^{2}f\left({x}_{0},{\mu }_{0}\right)\left(v,v\right)\right]=\frac{2{\left(\delta +\omega \right)}^{2}[\left(\pi {k}_{1}+{\theta }^{*}{k}_{2}\right)\left(\delta +\omega \right)\left(\lambda +\eta \right)-p{k}_{2}{\theta }^{*}\left(\lambda +\eta \right)-{k}_{1}\pi q(\delta +\omega )]}{p{k}_{1}{k}_{2}{{\theta }^{*}}^{2}(\lambda +\eta )}\ne 0$$

d)

$$ w^{T} \left[ {D^{3} f\left( {x_{0} ,\mu_{0} } \right)\left( {v,v, v} \right)} \right] \ne 0 $$

By Sotomayor theorem, this tells us that model (3–7) do not exhibit pitchfork bifurcation.

##### **Theorem 4**

Assume that ${\mathrm{R}}_{0}$ passes through the thresh hold value ${\mathrm{R}}_{0}=1.$ Then model [1–5] near the disease-free equilibrium point ${E}_{0}\left({L}_{h},{L}_{i},{v, B}_{h}, {B}_{i}\right)=\left({k}_{1},\mathrm{0,0}, {k}_{2}, 0\right)$ has:

1. Back ward bifurcation if $\frac{\delta +\omega }{p}-1<0,\frac{{\left(\lambda +\eta \right)}^{3}}{q{\pi }^{2}}-1<0\, and\, {v}_{2}>0$
2. Forward bifurcation if $\frac{\delta +\omega }{p}-1<0,\frac{{\left(\lambda +\eta \right)}^{3}}{q{\pi }^{2}}-1>0\, and\, {v}_{2}>0$

##### *Proof*

For simplicity, let ${x}_{1}={L}_{h}, {x}_{2}={L}_{i}, {x}_{3}=v, {x}_{4}={B}_{h}\, and\, {x}_{5}={B}_{i}$. Then model [1–5] is transformed to:

$$\frac{d{x}_{1}}{dt}=\sigma \left[1-\frac{{x}_{1}+{x}_{2}}{{k}_{1}}\right]{x}_{1}+\omega {x}_{2}-\frac{\theta {x}_{1}{x}_{3}}{{x}_{1}+{x}_{3}}={f}_{1}$$

$$\frac{d{x}_{2}}{dt}=\frac{\theta {x}_{1}{x}_{3}}{{x}_{1}+{x}_{3}}-\left(\delta +\omega \right){x}_{2}={f}_{2}$$

$$\frac{d{x}_{3}}{dt}=p{x}_{2}+q{x}_{5}-\left[\mu +\frac{\theta {x}_{1}}{{x}_{1}+{x}_{3}}+\frac{\pi {x}_{4}}{{x}_{4}+{x}_{3}}\right]{x}_{3}={f}_{3}$$

$$\frac{d{x}_{4}}{dt}=\psi \left[1-\frac{{x}_{4}+{x}_{5}}{{k}_{2}}\right]{x}_{4}+\lambda {x}_{5}-\frac{\pi {x}_{3}{x}_{4}}{{x}_{4}+{x}_{3}}={f}_{4}$$

$$\frac{d{x}_{5}}{dt}=\frac{\pi {x}_{3}{x}_{4}}{{x}_{4}+{x}_{3}}-\left(\lambda +\eta \right){x}_{5}={f}_{5}$$

Now, $$Df\left({x}_{0}, {\mu }_{0}\right)=\left[\begin{array}{cc}\begin{array}{c}-\sigma \\ \begin{array}{c}0\\ \begin{array}{c}0\\ \begin{array}{c}0\\ 0\end{array}\end{array}\end{array}\end{array}& \begin{array}{cc}\begin{array}{c}\omega -\sigma \\ \begin{array}{c}-\left(\delta +\omega \right)\\ \begin{array}{c}p\\ \begin{array}{c}0\\ 0\end{array}\end{array}\end{array}\end{array}& \begin{array}{cc}\begin{array}{c}\begin{array}{c}\begin{array}{c}-{\theta }^{*}\\ {\theta }^{*}\end{array}\\ -\left(\mu +{\theta }^{*}+\pi \right)\end{array}\\ \begin{array}{c}-\pi \\ \pi \end{array}\end{array}& \begin{array}{cc}\begin{array}{c}\begin{array}{c}\begin{array}{c}0\\ 0\end{array}\\ 0\end{array}\\ \begin{array}{c}-\psi \\ 0\end{array}\end{array}& \begin{array}{c}\begin{array}{c}\begin{array}{c}0\\ 0\end{array}\\ q\end{array}\\ \begin{array}{c}\lambda -\psi \\ -\left(\lambda +\eta \right)\end{array}\end{array}\end{array}\end{array}\end{array}\end{array}\right]=A$$

Let $W={\left({w}_{1},{w}_{2}, {w}_{3}, {w}_{4}, w{}_{5}\right)}^{T}$ be the right eigenvector of matrix A and $V=({v}_{1}, {v}_{2}, {v}_{3}, {v}_{4}, {v}_{5})$ be a left eigenvector of A which are corresponding to eigenvalue zero and $V.{W}^{T}=1$. Then,

$$\left({v}_{1} {v}_{2} {v}_{3} {v}_{4} {v}_{5}\right)\left[\begin{array}{cc}\begin{array}{c}-\sigma \\ \begin{array}{c}0\\ \begin{array}{c}0\\ \begin{array}{c}0\\ 0\end{array}\end{array}\end{array}\end{array}& \begin{array}{cc}\begin{array}{c}\omega -\sigma \\ \begin{array}{c}-\left(\delta +\omega \right)\\ \begin{array}{c}p\\ \begin{array}{c}0\\ 0\end{array}\end{array}\end{array}\end{array}& \begin{array}{cc}\begin{array}{c}\begin{array}{c}\begin{array}{c}-{\theta }^{*}\\ {\theta }^{*}\end{array}\\ -\left(\mu +{\theta }^{*}+\pi \right)\end{array}\\ \begin{array}{c}-\pi \\ \pi \end{array}\end{array}& \begin{array}{cc}\begin{array}{c}\begin{array}{c}\begin{array}{c}0\\ 0\end{array}\\ 0\end{array}\\ \begin{array}{c}-\psi \\ 0\end{array}\end{array}& \begin{array}{c}\begin{array}{c}\begin{array}{c}0\\ 0\end{array}\\ q\end{array}\\ \begin{array}{c}\lambda -\psi \\ -\left(\lambda +\eta \right)\end{array}\end{array}\end{array}\end{array}\end{array}\end{array}\right]=\left[\begin{array}{c}0\\ 0\\ \begin{array}{c}0\\ 0\\ 0\end{array}\end{array}\right]$$

$$\Rightarrow \left\{\begin{array}{c}-\sigma {v}_{1}=0\dots (a)\\ \left(\omega -\sigma \right){v}_{1}-\left(\delta +\omega \right){v}_{2}+p{v}_{3}=0\dots (b)\\ \begin{array}{c}-{\theta }^{*}{v}_{1}+{\theta }^{*}{v}_{2}-\left(\mu +{\theta }^{*}+\pi \right){v}_{3}-\pi {v}_{4}+\pi {v}_{5}=0\dots (c)\\ -\psi {v}_{4}=0\dots (d)\\ q{v}_{3}+\left(\lambda -\psi \right){v}_{4}-\left(\lambda +\eta \right){v}_{5}=0\dots (e)\end{array}\end{array}\right.$$

From (a) and (d), we have ${v}_{1}={v}_{4}=0$. Also, from (b) we have ${v}_{3}=\frac{\delta +\omega }{p}{v}_{2};$ letting ${v}_{2}$ free. From (c) and (e) we have: ${v}_{5}=\frac{q\left(\delta +\omega \right)}{p(\lambda +\eta )}{v}_{2}=\frac{\left(\mu +{\theta }^{*}+\pi \right)\left(\delta +\omega \right)-p{\theta }^{*}}{p\pi }{v}_{2}$.

$\Rightarrow V=(0, {v}_{2}, \frac{\delta +\omega }{p}{v}_{2}, 0, \frac{q\left(\delta +\omega \right)}{p\left(\lambda +\eta \right)}{v}_{2})$ . On the other hand $AW=0\boldsymbol{ }\,and\, V.W=1$

$$\Rightarrow \left[\begin{array}{cc}\begin{array}{c}-\sigma \\ \begin{array}{c}0\\ \begin{array}{c}0\\ \begin{array}{c}0\\ 0\end{array}\end{array}\end{array}\end{array}& \begin{array}{cc}\begin{array}{c}\omega -\sigma \\ \begin{array}{c}-\left(\delta +\omega \right)\\ \begin{array}{c}p\\ \begin{array}{c}0\\ 0\end{array}\end{array}\end{array}\end{array}& \begin{array}{cc}\begin{array}{c}\begin{array}{c}\begin{array}{c}-{\theta }^{*}\\ {\theta }^{*}\end{array}\\ -\left(\mu +{\theta }^{*}+\pi \right)\end{array}\\ \begin{array}{c}-\pi \\ \pi \end{array}\end{array}& \begin{array}{cc}\begin{array}{c}\begin{array}{c}\begin{array}{c}0\\ 0\end{array}\\ 0\end{array}\\ \begin{array}{c}-\psi \\ 0\end{array}\end{array}& \begin{array}{c}\begin{array}{c}\begin{array}{c}0\\ 0\end{array}\\ q\end{array}\\ \begin{array}{c}\lambda -\psi \\ -\left(\lambda +\eta \right)\end{array}\end{array}\end{array}\end{array}\end{array}\end{array}\right]\left[\begin{array}{c}{w}_{1}\\ {w}_{2}\\ \begin{array}{c}{w}_{3}\\ {w}_{4}\\ {w}_{5}\end{array}\end{array}\right]=\left[\begin{array}{c}0\\ 0\\ \begin{array}{c}0\\ 0\\ 0\end{array}\end{array}\right]$$

$$\Rightarrow \left\{\begin{array}{c}-\sigma {w}_{1}+\left(\omega -\sigma \right){w}_{2}-{\theta }^{*}{w}_{3}=0\dots (a)\\ -\left(\delta +\omega \right){w}_{2}+{\theta }^{*}{w}_{3}=0\dots (b)\\ \begin{array}{c}p{w}_{2}-\left(\mu +\pi +{\theta }^{*}\right){w}_{3}+q{w}_{5}=0\dots (c)\\ -\pi {w}_{3}-\psi {w}_{4}+\left(\lambda -\psi \right){w}_{5}=0\dots (d)\\ \pi {w}_{3}-\left(\lambda +\eta \right){w}_{5}=0\dots (e)\end{array}\end{array}\right.$$

Letting ${w}_{2}$ free, we have ${w}_{1}=-\frac{\left(\sigma +\delta \right)}{\sigma }{w}_{2}, \frac{\delta +\omega }{{\theta }^{*}}{w}_{2}, {w}_{4}=-\frac{\pi \left(\delta +\omega \right)\left(\psi +\eta \right)}{\psi {\theta }^{*}\left(\lambda +\eta \right)} {w}_{2}, {w}_{5}=\frac{\pi \left(\delta +\omega \right)}{{\theta }^{*}\left(\lambda +\eta \right)}{w}_{2}=\frac{\left(\left(\mu +{\theta }^{*}+\pi \right)\left(\delta +\omega \right)-p{\theta }^{*}\right)}{{\theta }^{*}q}{w}_{2}$

$$\Rightarrow {W}^{T}=(-\frac{\left(\sigma +\delta \right)}{\sigma }{w}_{2}, {w}_{2}, \frac{\delta +\omega }{{\theta }^{*}}{w}_{2}, -\frac{\pi \left(\delta +\omega \right)\left(\psi +\eta \right)}{\psi {\theta }^{*}\left(\lambda +\eta \right)} {w}_{2}, \frac{\pi \left(\delta +\omega \right)}{{\theta }^{*}(\lambda +\eta )}{w}_{2})$$

Now, we choose ${v}_{2}\, and\, {w}_{2}$ such that $V.{W}^{T}=1$

$$\Leftrightarrow \left(0, {v}_{2}, \frac{\delta +\omega }{p}{v}_{2}, 0, \frac{q\left(\delta +\omega \right)}{p\left(\lambda +\eta \right)}{v}_{2}\right).\left(-\frac{\left(\sigma +\delta \right)}{\sigma }{w}_{2}, {w}_{2}, \frac{\delta +\omega }{{\theta }^{*}}{w}_{2}, -\frac{\pi \left(\delta +\omega \right)\left(\psi +\eta \right)}{\psi {\theta }^{*}\left(\lambda +\eta \right)} {w}_{2}, \frac{\pi \left(\delta +\omega \right)}{{\theta }^{*}\left(\lambda +\eta \right)}{w}_{2}\right)=1$$

$$\Leftrightarrow 0+{v}_{2}{w}_{2}+\frac{{\left(\delta +\omega \right)}^{2}}{p{\theta }^{*}}{v}_{2}{w}_{2}+0+\frac{q\pi {\left(\delta +\omega \right)}^{2}}{{p\theta }^{*}{\left(\lambda +\eta \right)}^{2}}{v}_{2}{w}_{2}=1$$

$$\Leftrightarrow [1+\frac{{\left(\delta +\omega \right)}^{2}}{p{\theta }^{*}}+\frac{q\pi {\left(\delta +\omega \right)}^{2}}{{p\theta }^{*}{\left(\lambda +\eta \right)}^{2}}]{v}_{2}{w}_{2}=1$$

$$\Leftrightarrow {v}_{2}{w}_{2}=\frac{1}{\left[1+\frac{{\left(\delta +\omega \right)}^{2}}{p{\theta }^{*}}+\frac{q\pi {\left(\delta +\omega \right)}^{2}}{{p\theta }^{*}{\left(\lambda +\eta \right)}^{2}}\right]}>0$$

Thus, we should choose ${v}_{2}$ and ${w}_{2}$ which have the same sign. That is, we can choose ${v}_{2}<0$ and ${w}_{2}<0$ or ${v}_{2}>0$ and ${w}_{2}>0$.

Now, $$a=\sum_{k,i,j=1}^{n}{v}_{k}{w}_{i}{w}_{j}\frac{{\partial }^{2}{f}_{k}}{\partial {x}_{i}\partial {x}_{j}}({x}_{0},{\mu }_{0}) \,and\, b=\sum_{k,i,j=1}^{n}{v}_{k}{w}_{i}\frac{{\partial }^{2}{f}_{k}}{\partial {x}_{i}\partial \mu }({x}_{0},{\mu }_{0})$$

(A) Calculation of ${v}_{k}{w}_{i}{w}_{j}\frac{{\partial }^{2}{f}_{k}}{\partial {x}_{i}\partial {x}_{j}}({x}_{0},{\mu }_{0})$ is as follows:

$${v}_{k}{w}_{i}{w}_{j}\frac{{\partial }^{2}{f}_{k}}{\partial {x}_{i}\partial {x}_{j}}\left({x}_{0},{\mu }_{0}\right)=0$$ for $$k=1 and k= 4$$, since $${v}_{1}={v}_{4}=0$$.

For k = 2, we have:

$${v}_{2}{w}_{1}^{2}\frac{{\partial }^{2}{f}_{2}}{\partial {x}_{1}^{2}}\left({x}_{0},{\mu }_{0}\right)= 0,{v}_{2}{w}_{1}{w}_{2}\frac{{\partial }^{2}{f}_{2}}{\partial {x}_{1}\partial {x}_{2}}\left({x}_{0},{\mu }_{0}\right)= 0, {v}_{2}{w}_{1}{w}_{3}\frac{{\partial }^{2}{f}_{2}}{\partial {x}_{1}\partial {x}_{3}}\left({x}_{0},{\mu }_{0}\right)= 0$$

$${v}_{2}{w}_{1}{w}_{4}\frac{{\partial }^{2}{f}_{2}}{\partial {x}_{1}\partial {x}_{4}}\left({x}_{0},{\mu }_{0}\right)= 0, {v}_{2}{w}_{1}{w}_{5}\frac{{\partial }^{2}{f}_{2}}{\partial {x}_{1}\partial {x}_{5}}\left({x}_{0},{\mu }_{0}\right)= 0,{v}_{2}{w}_{2}^{2}\frac{{\partial }^{2}{f}_{2}}{\partial {x}_{2}^{2}}\left({x}_{0},{\mu }_{0}\right)=0,$$

$${v}_{2}{w}_{2}{w}_{3}\frac{{\partial }^{2}{f}_{2}}{\partial {x}_{2}\partial {x}_{3}}\left({x}_{0},{\mu }_{0}\right)= 0,{v}_{2}{w}_{2}{w}_{4}\frac{{\partial }^{2}{f}_{2}}{\partial {x}_{2}\partial {x}_{4}}\left({x}_{0},{\mu }_{0}\right)= 0, {v}_{2}{w}_{2}{w}_{5}\frac{{\partial }^{2}{f}_{2}}{\partial {x}_{2}\partial {x}_{5}}\left({x}_{0},{\mu }_{0}\right)= 0,$$

$${v}_{2}{w}_{3}^{2}\frac{{\partial }^{2}{f}_{2}}{\partial {x}_{3}^{2}}\left({x}_{0},{\mu }_{0}\right)=-\frac{2{\left(\delta +\omega \right)}^{2}}{{k}_{1}{\theta }^{*}}{{v}_{2}{w}_{2}}^{2},{v}_{2}{w}_{3}{w}_{4}\frac{{\partial }^{2}{f}_{2}}{\partial {x}_{3}\partial {x}_{4}}\left({x}_{0},{\mu }_{0}\right)= 0,$$

$${v}_{2}{w}_{3}{w}_{5}\frac{{\partial }^{2}{f}_{2}}{\partial {x}_{3}\partial {x}_{5}}\left({x}_{0},{\mu }_{0}\right)= 0 ,{v}_{2}{w}_{4}^{2}\frac{{\partial }^{2}{f}_{2}}{\partial {x}_{4}^{2}}\left({x}_{0},{\mu }_{0}\right)=0,{v}_{2}{w}_{4}{w}_{5}\frac{{\partial }^{2}{f}_{2}}{\partial {x}_{4}\partial {x}_{5}}\left({x}_{0},{\mu }_{0}\right)= 0,$$

$${v}_{2}{w}_{5}^{2}\frac{{\partial }^{2}{f}_{2}}{\partial {x}_{5}^{2}}\left({x}_{0},{\mu }_{0}\right)=0.$$

For k = 3, we have:

$${v}_{3}{w}_{1}^{2}\frac{{\partial }^{2}{f}_{3}}{\partial {x}_{1}^{2}}\left({x}_{0},{\mu }_{0}\right)=0, {v}_{3}{w}_{1}{w}_{2}\frac{{\partial }^{2}{f}_{3}}{\partial {x}_{1}\partial {x}_{2}}\left({x}_{0},{\mu }_{0}\right)= 0, {v}_{3}{w}_{1}{w}_{3}\frac{{\partial }^{2}3}{\partial {x}_{1}\partial {x}_{3}}\left({x}_{0},{\mu }_{0}\right)= 0,$$

$${v}_{3}{w}_{1}{w}_{4}\frac{{\partial }^{2}{f}_{3}}{\partial {x}_{1}\partial {x}_{4}}\left({x}_{0},{\mu }_{0}\right)= 0, {v}_{3}{w}_{1}{w}_{5}\frac{{\partial }^{2}{f}_{3}}{\partial {x}_{1}\partial {x}_{5}}\left({x}_{0},{\mu }_{0}\right)= 0, {v}_{3}{w}_{2}^{2}\frac{{\partial }^{2}{f}_{3}}{\partial {x}_{2}^{2}}\left({x}_{0},{\mu }_{0}\right)=0,$$

$${v}_{3}{w}_{2}{w}_{3}\frac{{\partial }^{2}{f}_{3}}{\partial {x}_{2}\partial {x}_{3}}\left({x}_{0},{\mu }_{0}\right)= 0,{v}_{3}{w}_{2}{w}_{4}\frac{{\partial }^{2}{f}_{3}}{\partial {x}_{2}\partial {x}_{4}}\left({x}_{0},{\mu }_{0}\right)= 0, {v}_{3}{w}_{2}{w}_{5}\frac{{\partial }^{2}{f}_{3}}{\partial {x}_{2}\partial {x}_{5}}\left({x}_{0},{\mu }_{0}\right)= 0,$$

$${v}_{3}{w}_{3}^{2}\frac{{\partial }^{2}{f}_{3}}{\partial {x}_{3}^{2}}\left({x}_{0},{\mu }_{0}\right)=2\left[\frac{{\theta }^{*}}{{k}_{1}}+\frac{\pi }{{k}_{2}}\right]\frac{{\left(\delta +\omega \right)}^{3}}{p{{\theta }^{*}}^{2}} {v}_{2}{w}_{2}^{2},{v}_{3}{w}_{3}{w}_{4}\frac{{\partial }^{2}{f}_{3}}{\partial {x}_{3}\partial {x}_{4}}\left({x}_{0},{\mu }_{0}\right)= 0,$$

$${v}_{3}{w}_{3}{w}_{5}\frac{{\partial }^{2}{f}_{3}}{\partial {x}_{3}\partial {x}_{5}}\left({x}_{0},{\mu }_{0}\right)= 0,{v}_{3}{w}_{3}{w}_{5}\frac{{\partial }^{2}{f}_{3}}{\partial {x}_{3}\partial {x}_{5}}\left({x}_{0},{\mu }_{0}\right)= 0,{v}_{3}{w}_{4}^{2}\frac{{\partial }^{2}{f}_{3}}{\partial {x}_{4}^{2}}\left({x}_{0},{\mu }_{0}\right)=0,$$

$${v}_{3}{w}_{4}{w}_{5}\frac{{\partial }^{2}{f}_{3}}{\partial {x}_{4}\partial {x}_{5}}\left({x}_{0},{\mu }_{0}\right)= 0, {v}_{3}{w}_{5}^{2}\frac{{\partial }^{2}{f}_{3}}{\partial {x}_{5}^{2}}\left({x}_{0},{\mu }_{0}\right)=0.$$

For k = 5, we get:

$${v}_{5}{w}_{1}^{2}\frac{{\partial }^{2}{f}_{5}}{\partial {x}_{1}^{2}}\left({x}_{0},{\mu }_{0}\right)= 0,{v}_{5}{w}_{1}{w}_{2}\frac{{\partial }^{2}{f}_{5}}{\partial {x}_{1}\partial {x}_{2}}\left({x}_{0},{\mu }_{0}\right)= 0, {v}_{5}{w}_{1}{w}_{3}\frac{{\partial }^{2}{f}_{5}}{\partial {x}_{1}\partial {x}_{3}}\left({x}_{0},{\mu }_{0}\right)= 0$$

$${v}_{5}{w}_{1}{w}_{4}\frac{{\partial }^{2}{f}_{5}}{\partial {x}_{1}\partial {x}_{4}}\left({x}_{0},{\mu }_{0}\right)= 0, {v}_{5}{w}_{1}{w}_{5}\frac{{\partial }^{2}{f}_{5}}{\partial {x}_{1}\partial {x}_{5}}\left({x}_{0},{\mu }_{0}\right)= 0,{v}_{5}{w}_{2}^{2}\frac{{\partial }^{2}{f}_{5}}{\partial {x}_{2}^{2}}\left({x}_{0},{\mu }_{0}\right)=0,$$

$${v}_{5}{w}_{2}{w}_{3}\frac{{\partial }^{2}{f}_{5}}{\partial {x}_{2}\partial {x}_{3}}\left({x}_{0},{\mu }_{0}\right)= 0,{v}_{5}{w}_{2}{w}_{4}\frac{{\partial }^{2}{f}_{5}}{\partial {x}_{2}\partial {x}_{4}}\left({x}_{0},{\mu }_{0}\right)= 0, {v}_{5}{w}_{2}{w}_{5}\frac{{\partial }^{2}{f}_{5}}{\partial {x}_{2}\partial {x}_{5}}\left({x}_{0},{\mu }_{0}\right)= 0,$$

$${v}_{5}{w}_{3}^{2}\frac{{\partial }^{2}{f}_{5}}{\partial {x}_{3}^{2}}\left({x}_{0},{\mu }_{0}\right)=-\frac{2q{\pi }^{3}{\left(\delta +\omega \right)}^{3}}{p{k}_{2}{{\theta }^{*}}^{2}{\left(\lambda +\eta \right)}^{3}}{v}_{2}{w}_{2}^{2},{v}_{5}{w}_{3}{w}_{4}\frac{{\partial }^{2}{f}_{5}}{\partial {x}_{3}\partial {x}_{4}}\left({x}_{0},{\mu }_{0}\right)=0,$$

$${v}_{5}{w}_{3}{w}_{5}\frac{{\partial }^{2}{f}_{5}}{\partial {x}_{3}\partial {x}_{5}}\left({x}_{0},{\mu }_{0}\right)= 0, {v}_{5}{w}_{4}^{2}\frac{{\partial }^{2}{f}_{5}}{\partial {x}_{4}^{2}}\left({x}_{0},{\mu }_{0}\right)=0,{v}_{5}{w}_{4}{w}_{5}\frac{{\partial }^{2}{f}_{5}}{\partial {x}_{4}\partial {x}_{5}}\left({x}_{0},{\mu }_{0}\right)= 0,$$

$${v}_{5}{w}_{5}^{2}\frac{{\partial }^{2}{f}_{5}}{\partial {x}_{5}^{2}}\left({x}_{0},{\mu }_{0}\right)=0.$$

Thus, $$a=\sum_{k,i,j=1}^{n}{v}_{k}{w}_{i}{w}_{j}\frac{{\partial }^{2}{f}_{k}}{\partial {x}_{i}\partial {x}_{j}}\left({x}_{0},{\mu }_{0}\right)=\left\{-\frac{2{\left(\delta +\omega \right)}^{2}}{{k}_{1}{\theta }^{*}}{{v}_{2}{w}_{2}}^{2}+2\left[\frac{{\theta }^{*}}{{k}_{1}}+\frac{\pi }{{k}_{2}}\right]\frac{{\left(\delta +\omega \right)}^{3}}{p{{\theta }^{*}}^{2}} {v}_{2}{w}_{2}^{2} -\frac{2q{\pi }^{3}{\left(\delta +\omega \right)}^{3}}{p{k}_{2}{{\theta }^{*}}^{2}{\left(\lambda +\eta \right)}^{3}}{v}_{2}{w}_{2}^{2}\right\}$$

$$\Rightarrow a=\frac{2{\left(\delta +\omega \right)}^{2}}{{\theta }^{*}}[-\frac{1}{{k}_{1}}+\left[\frac{{\theta }^{*}}{{k}_{1}}+\frac{\pi }{{k}_{2}}\right]\frac{\left(\delta +\omega \right)}{p{\theta }^{*}}-\frac{q{\pi }^{3}(\delta +\omega )}{p{k}_{2}{\theta }^{*}{\left(\lambda +\eta \right)}^{3}}]{{v}_{2}{w}_{2}}^{2}$$

$\Rightarrow a=\frac{2{\left(\delta +\omega \right)}^{2}}{p{\theta }^{*}}[\frac{\delta +\omega -p}{{k}_{1}}+\frac{\pi \left(\delta +\omega \right)[{\left(\lambda +\eta \right)}^{3}-q{\pi }^{2}]}{{k}_{2}{\theta }^{*}{\left(\lambda +\eta \right)}^{3}}]{{v}_{2}{w}_{2}}^{2}$ . Here, the value of $a$ is depending on the value of $(\delta +\omega )-p, {\left(\lambda +\eta \right)}^{3}-q{\pi }^{2} \,and\, {v}_{2};$ because for every ${w}_{2}\ne o, {w}_{2}^{2}>0.$

Hence;

**Case 1:** If $\frac{\delta +\omega }{p}-1>0,\frac{{\left(\lambda +\eta \right)}^{3}}{q{\pi }^{2}}-1>0\, and\, {v}_{2}>0,$ then $a>0$

**Case 2:** If $\frac{\delta +\omega }{p}-1<0,\frac{{\left(\lambda +\eta \right)}^{3}}{q{\pi }^{2}}-1<0 \, and\, {v}_{2}>0,$ then $a>0$

**Case 3:** If $\frac{\delta +\omega }{p}-1>0,\frac{{\left(\lambda +\eta \right)}^{3}}{q{\pi }^{2}}-1>0 \, and\, {v}_{2}<0,$ then $a<0$

**Case 4:** If $\frac{\delta +\omega }{p}-1<0,\frac{{\left(\lambda +\eta \right)}^{3}}{q{\pi }^{2}}-1>0 \, and\, {v}_{2}>0,$ then $a<0$

**Case 5:** If $\frac{\delta +\omega }{p}-1>0,\frac{{\left(\lambda +\eta \right)}^{3}}{q{\pi }^{2}}-1<0 \, and\, {v}_{2}<0,$ then $a<0$

(B) From calculation of ${v}_{k}{w}_{i}\frac{{\partial }^{2}{f}_{k}}{\partial {x}_{i}\partial {\theta }^{*}}({x}_{0},{\mu }_{0})$ , we have:

$${v}_{k}{w}_{i}\frac{{\partial }^{2}{f}_{k}}{\partial {x}_{i}\partial \mu }\left({x}_{0},{\mu }_{0}\right)=0$$ for $$k=1 and k= 4$$, since $${v}_{1}={v}_{4}=0$$.

For k = 2, we have:

$$ v_{2} w_{1} \frac{{\partial^{2} f_{2} }}{{\partial x_{1} \partial \theta^{*} }} = 0, v_{2} w_{2} \frac{{\partial^{2} f_{2} }}{{\partial x_{2} \partial \theta^{*} }} = 0, v_{2} w_{3} \frac{{\partial^{2} f_{2} }}{{\partial x_{3} \partial \theta^{*} }} = \frac{\delta + \omega }{{\theta^{*} }}v_{2} w_{2} , v_{2} w_{4} \frac{{\partial^{2} f_{2} }}{{\partial x_{4} \partial \theta^{*} }} = 0, v_{2} w_{5} \frac{{\partial^{2} f_{2} }}{{\partial x_{5} \partial \theta^{*} }} = 0 $$

$$ v_{2} w_{4} \frac{{\partial^{2} f_{2} }}{{\partial x_{4} \partial \theta^{*} }} = 0, v_{2} w_{5} \frac{{\partial^{2} f_{2} }}{{\partial x_{5} \partial \theta^{*} }} = 0. $$

For k = 3, we have:

$${v}_{3}{w}_{1}\frac{{\partial }^{2}{f}_{3}}{\partial {x}_{1}\partial {\theta }^{*}}=0, {v}_{3}{w}_{2}\frac{{\partial }^{2}{f}_{3}}{\partial {x}_{2}\partial {\theta }^{*}}=0, {v}_{3}{w}_{3}\frac{{\partial }^{2}{f}_{3}}{\partial {x}_{3}\partial {\theta }^{*}}=-\frac{{\left(\delta +\omega \right)}^{2}}{p{\theta }^{*}}{v}_{2}{w}_{2}, {v}_{3}{w}_{4}\frac{{\partial }^{2}{f}_{3}}{\partial {x}_{4}\partial {\theta }^{*}}=0, {v}_{3}{w}_{5}\frac{{\partial }^{2}{f}_{3}}{\partial {x}_{5}\partial {\theta }^{*}}=0$$

For k = 5, we get:

$$ v_{5} w_{1} \frac{{\partial^{2} f_{5} }}{{\partial x_{1} \partial \theta^{*} }} = 0, v_{5} w_{2} \frac{{\partial^{2} f_{5} }}{{\partial x_{2} \partial \theta^{*} }} = 0, v_{5} w_{3} \frac{{\partial^{2} f_{5} }}{{\partial x_{3} \partial \theta^{*} }} = 0,v_{5} w_{4} \frac{{\partial^{2} f_{5} }}{{\partial x_{4} \partial \theta^{*} }} = 0, v_{5} w_{5} \frac{{\partial^{2} f_{5} }}{{\partial x_{5} \partial \theta^{*} }} = 0. $$

Therefore, $$b=\sum_{k,i,j=1}^{n}{v}_{k}{w}_{i}\frac{{\partial }^{2}{f}_{k}}{\partial {x}_{i}\partial \mu }\left({x}_{0},{\mu }_{0}\right)=\frac{\delta +\omega }{{\theta }^{*}}{v}_{2}{w}_{2}-\frac{{\left(\delta +\omega \right)}^{2}}{p{\theta }^{*}}{v}_{2}{w}_{2}$$

$\Rightarrow b=\frac{\left(\delta +\omega \right)}{{\theta }^{*}}\left[1-\frac{\left(\delta +\omega \right)}{p}\right]{v}_{2}{w}_{2}$. Here, the value of $b$ depends on the value of $1-\frac{\left(\delta +\omega \right)}{p}\, because\, {v}_{2}{w}_{2}>0$.

**Case 1:** If $\frac{\left(\delta +\omega \right)}{p}-1>0 ,$ then $b<0.$

**Case 2:** If $\frac{\left(\delta +\omega \right)}{p}-1<0 ,$ then $b>0.$

From (A) and (B), we found that $a=\frac{2{\left(\delta +\omega \right)}^{2}}{p{\theta }^{*}}[\frac{\delta +\omega -p}{{k}_{1}}+\frac{\pi \left(\delta +\omega \right)[{\left(\lambda +\eta \right)}^{2}-q{\pi }^{2}]}{{k}_{2}{\theta }^{*}{\left(\lambda +\eta \right)}^{3}}]{{v}_{2}{w}_{2}}^{2}\, and\, b=\frac{\left(\delta +\omega \right)}{{\theta }^{*}}\left[1-\frac{\left(\delta +\omega \right)}{p}\right]{v}_{2}{w}_{2}$.

Thus, if $\frac{\delta +\omega }{p}-1<0,\frac{{\left(\lambda +\eta \right)}^{3}}{q{\pi }^{2}}-1<0\, and\, {v}_{2}>0,$ then both $a\, and\, b$ are greater than zero; and the model exhibits backward bifurcation; and if $\frac{\delta +\omega }{p}-1<0,\frac{{\left(\lambda +\eta \right)}^{3}}{q{\pi }^{2}}-1>0 and {v}_{2}>0$, then a is less than zero and b is greater than zero and the model exhibits forward bifurcation.

## Discussion

From the dynamical model of bifurcation analysis with non-Cytolytic cure processes of HBV on infected liver and blood cells, we have seen that the model exhibits back ward bifurcation if $\delta +\omega <p, {\left(\lambda +\eta \right)}^{3}<q{\pi }^{2}\, and\, {v}_{2}>0$. This means, whenever the sum of death rate of infected liver cells and rate of cure of infected liver cells by non-Cytolytic cure process is less than rate of release of free viruses by an infected liver cell; the cube of sum of rate of cure of infected blood cells by non-Cytolytic cure process and death rate of infected blood cells is less than product of square of rate of infection of blood cell by free viruses by the rate of release of free viruses with an infected blood cell and ${v}_{2}>0;$ then there is backward bifurcation or relapse or reinfection of HBV occurs in the host. On the other hand, if $\delta +\omega <p, {\left(\lambda +\eta \right)}^{3}>q{\pi }^{2}\, and\, {v}_{2}>0$, then the dynamical model exhibits forward bifurcation. This means, whenever the sum of death rate of infected liver cells and rate of cure of infected liver cells by non-Cytolytic cure process is less than rate of release of free viruses by an infected liver cell; the cube of sum of rate of cure of infected blood cells by non-Cytolytic cure process and death rate of infected blood cells is greater than product of square of rate of infection of blood cell by free viruses by the rate of release of free viruses with an infected blood cell and ${v}_{2}>0;$ then there is forward bifurcation or there is no relapse or reinfection of HBV occurs in the host.

## Conclusion

In this paper, a mathematical model for HBV that explores the interaction of viral particles in both the liver and the blood is developed. I have found that the HBV with non-Cytolytic cure process on infected liver and blood cells model is well-posed and useful for the description of hepatitis infection dynamics. The model has both a disease-free equilibrium and endemic state, similar to the basic models. Assuming that ${\mathrm{R}}_{0}$ passes through the value ${\mathrm{R}}_{0}=1;$ the model near the disease-free equilibrium ${E}_{0}\left({L}_{h},{L}_{i},{v, B}_{h}, {B}_{i}\right)=\left({k}_{1},\mathrm{0,0}, {k}_{2}, 0\right)$ has a trans- critical bifurcation but has no saddle-node and pitchfork bifurcation. However, a backward bifurcation can take place if $\delta +\omega <p, {\left(\lambda +\eta \right)}^{3}<q{\pi }^{2}\, and\, {v}_{2}>0$. The existence of a backward bifurcation is an interesting artifact since this means that the disease cannot be eradicated by simply reducing the value of the basic reproduction number ${R}_{0}$ below 1.This can have important implications on drug therapy protocols, since it sheds light on possible control mechanisms for disease eradication. If $\delta +\omega <p, {\left(\lambda +\eta \right)}^{3}>q{\pi }^{2}\, and\, {v}_{2}>0$, then the model exhibits forward bifurcation. This tell us that in the case of transplantation of HBV chronically infected liver, reinfection or relapse is not occur.

## Data Availability

The datasets used and/or analyzed during the current study are available from the corresponding author on reasonable request.
